# Improved kinetic behaviour of Mg(NH_2_)_2_-2LiH doped with nanostructured K-modified-Li_x_Ti_y_O_z_ for hydrogen storage

**DOI:** 10.1038/s41598-019-55770-y

**Published:** 2020-01-07

**Authors:** Gökhan Gizer, Julián Puszkiel, Maria Victoria Castro Riglos, Claudio Pistidda, José Martín Ramallo-López, Martin Mizrahi, Antonio Santoru, Thomas Gemming, Jo-Chi Tseng, Thomas Klassen, Martin Dornheim

**Affiliations:** 1 Institute of Materials Research, Materials Technology, Helmholtz-Zentrum Geesthacht GmbH, Max-Planck Strasse 1, D-21502 Geesthacht, Germany; 2 Consejo Nacional de Investigaciones Científicas y Técnicas (CONICET), Centro Atómico Bariloche, Av. Bustillo km 9500, S.C. de Bariloche, Argentina; 3 Instituto de Investigaciones Fisicoquímicas Teóricas y Aplicadas, INIFTA (CCT La Plata-CONICET, UNLP), Diagonal 113 y Calle 64, 1900 La Plata, Argentina; 4 IFW Dresden, P.O. Box 270016, D-01171 Dresden, Germany; 5 Deutsches elektronen-Synchrotron, Notkestr. 85, 22607 Hamburg, Germany

**Keywords:** Materials science, Energy science and technology

## Abstract

The system Mg(NH_2_)_2_ + 2LiH is considered as an interesting solid-state hydrogen storage material owing to its low thermodynamic stability of ca. 40 kJ/mol H_2_ and high gravimetric hydrogen capacity of 5.6 wt.%. However, high kinetic barriers lead to slow absorption/desorption rates even at relatively high temperatures (>180 °C). In this work, we investigate the effects of the addition of K-modified Li_x_Ti_y_O_z_ on the absorption/desorption behaviour of the Mg(NH_2_)_2_ + 2LiH system. In comparison with the pristine Mg(NH_2_)_2_ + 2LiH, the system containing a tiny amount of nanostructured K-modified Li_x_Ti_y_O_z_ shows enhanced absorption/desorption behaviour. The doped material presents a sensibly reduced (∼30 °C) desorption onset temperature, notably shorter hydrogen absorption/desorption times and reversible hydrogen capacity of about 3 wt.% H_2_ upon cycling. Studies on the absorption/desorption processes and micro/nanostructural characterizations of the Mg(NH_2_)_2_ + 2LiH + K-modified Li_x_Ti_y_O_z_ system hint to the fact that the presence of *in situ* formed nanostructure K_2_TiO_3_ is the main responsible for the observed improved kinetic behaviour.

## Introduction

One of the limiting factors for the implementation of hydrogen in stationary and mobile applications is the lack of an efficient and safe storage system. For mobile applications, a fuel cell equipped electrical car requires about 5 kg of hydrogen in order to achieve a driving range of ca. 500 km^1^. However, storing 5 kg of hydrogen in a high-pressure (700 bar) tank requires an internal volume of 122 litres^2^. In order to improve the volumetric hydrogen storage capacity, solid-state storage in metal hydrides is considered as an effective approach^3–8^. As an example, excluding the volume of the tank material, 5 kg of hydrogen can be stored in magnesium hydride (MgH_2_) occupying a volume of only 46 litres^7^. However, due to its high desorption enthalpy (ΔH_*des*_ = 74 kJ mol^−1^), MgH_2_ requires high dehydrogenation temperatures (>300 °C)^9^. By the reaction of alkali metals (i.e. Na, K) with gaseous ammonia, metal amides (i.e. NaNH_2_ and KNH_2_) were firstly discovered at the beginning of 1800s^10,11^. During the last 50 years, metal amides were not considered as potential hydrogen storage materials since the detected main gaseous product from their thermal decomposition was ammonia^12,13^. In 2002, Chen *et al*. reported for the first time that a material composed of LiNH_2_ and LiH was able to reversibly store 6.5 wt. % of H_2_ at 255 °C^14^. Thereafter, a focus was given to synthesize and understand the mechanisms in amide-imide systems for hydrogen storage^15,16^. Replacing LiNH_2_ with Mg(NH_2_)_2_, a reversible H_2_ storage capacity of 5.5 wt. % at operating temperatures of 200 °C is obtained according to reactions (1) and (2) (ΔH_*des*_ = 40 kJ mol^−1^)^17^.

1 $$\,2{\rm{M}}{\rm{g}}{({{\rm{N}}{\rm{H}}}_{2})}_{2}+4{\rm{L}}{\rm{i}}{\rm{H}}\leftrightarrow {{\rm{L}}{\rm{i}}}_{2}{{\rm{M}}{\rm{g}}}_{2}{({\rm{N}}{\rm{H}})}_{3}+{{\rm{L}}{\rm{i}}{\rm{N}}{\rm{H}}}_{2}+{\rm{L}}{\rm{i}}{\rm{H}}+3{{\rm{H}}}_{2}$$

2 $${{\rm{Li}}}_{2}{{\rm{Mg}}}_{2}{({\rm{NH}})}_{3}+{{\rm{LiNH}}}_{2}+{\rm{LiH}}\leftrightarrow 2{{\rm{Li}}}_{2}{\rm{Mg}}{({\rm{NH}})}_{2}+{{\rm{H}}}_{2}$$

According to the calculated thermodynamic properties of the Mg(NH_2_)_2_ + 2LiH stoichiometric mixture, operating temperatures of 90 °C can be achieved at 1 bar^18^. However, sufficient dehydrogenation rates, even after intense ball milling treatment, can be obtained only at temperatures above 180 °C due to harsh kinetic constraints^19^. Several attempts have been made in order to improve the sluggish dehydrogenation behaviour of the Mg(NH_2_)_2_ + 2LiH composite system^18–39^. Potassium containing additives effectively reduce the dehydrogenation peak temperature down to 130 °C, which is ∼50 °C lower than that of pristine Mg(NH_2_)_2_ + 2LiH^40–42^. However, due to segregation phenomena that occurs at high-temperature (≥180 °C) upon cycling, the inhomogeneous distribution of the K-species reduces their catalytic activity^43^. Therefore, the design/synthesis of new additives is mandatory in order to achieve long-lasting absorption/desorption properties. TiO_2_ is one of the low-cost additives which enhance the hydrogen storage properties of the 2LiBH_4_ + MgH_2_ reactive hydride composite (RHC) system^44–46^. Puszkiel *et al*. showed that 2LiH + MgB_2_/2LiBH_4_ + MgH_2_ RHC system doped with core-shell Li_x_TiO_2_ nanoparticles shows improved the kinetic and cycling behaviour^44^. It was found that the core-shell Li_x_TiO_2_ nanoparticles act as Li^+^ pumps, increasing Li^+^ mobility, hence accounting for the observed enhanced hydrogen storage properties. Studies on reaction mechanism of Mg(NH_2_)_2_ + LiH system showed that diffusion of small ions (e.g., Li^+^, Mg^+2^, and H^+^) might account for the improved reaction kinetics^47–50^. In this work, we investigate the effect of Li_x_Ti_y_O_z_ and potassium-modified Li_x_Ti_y_O_z_ additives on the microstructural and hydrogen storage properties of Mg(NH_2_)_2_ + 2LiH system.

## Experimental

### Additive synthesis

All reagents utilized in this work were Mg(NH_2_)_2_ (described in the following subsection), LiH (Alfa Aesar, 97 % purity), anatase TiO_2_ (Sigma Aldrich, >99 % purity, - 325 mesh) and KH (Sigma Aldrich, suspension 35% in mineral oil). The investigated additives were obtained by milling LiH, TiO_2_ and KH in different stoichiometric ratios under argon atmosphere for two hours and then annealing them under Ar atmosphere at 600 °C for 8 hours. The stoichiometry of the reagent utilized to synthesize the additives were: 1) 0.5LiH + TiO_2_ and 2) 0.5LiH + TiO_2_ + 0.25KH. In addition to the prepared additives, KH alone was also used as an additive. In order to separate mineral oil from KH, three washing cycles in hexane were carried out. After that, hexane was removed by applying dynamic vacuum.

### Material synthesis

Mg(NH_2_)_2_ (95 % purity) was *in-house* synthesized by ball milling MgH_2_ under NH_3_ atmosphere, followed by annealing at 300 °C under NH_3_ atmosphere. The details of the synthesis were described in our previous study^50^. The Mg(NH_2_)_2_ was mixed with LiH (Alfa Aesar, 97 % purity) and 1.0, 2.5 or 5 mol. % of additives (Section 2.2). All materials were milled in a Fritsch P6 Planetary ball miller for 5 hours with ball to powder ratio 60:1 under 50 bar of H_2_ pressure. The sample names used to identify the prepared specimens are listed in Table 1.

**Table 1:** Compositions and designations for the investigated samples.

Sample composition	Sample code
Mg(NH_2_)_2_ + 2LiH	Mg-Li
Mg(NH_2_)_2_ + 2LiH + 0.05(0.5LiH + TiO_2_)	Mg-Li-5LTO
Mg(NH_2_)_2_ + 2LiH + 0.05(0.5LiH + TiO_2_ + 0.25KH)	Mg-Li-5LTOK
Mg(NH_2_)_2_ + 2LiH + 0.025(0.5LiH + TiO_2_ + 0.25KH)	Mg-Li-2.5LTOK
Mg(NH_2_)_2_ + 2LiH + 0.010(0.5LiH + TiO_2_ + 0.25KH)	Mg-Li-1LTOK
Mg(NH_2_)_2_ + 2LiH + 0.05KH	Mg-Li-5K

### Characterization techniques

*Ex situ* powder X-ray diffraction method (PXD) was applied for the identification of crystalline phases, by using a Bruker D8 Discover diffractometer equipped with Cu X-ray source (λ = 1.54 Å) operating at 50 kV and 1000 mA and a 2D VANTEC detector. Diffraction patterns were collected in the 2θ range 20° to 80°. A sample holder sealed with a polymethylmethacrylate (PMMA) dome was utilized to prevent the material oxidation during PXD measurements.

*In situ* synchrotron radiation powder X-ray diffraction (SR*-*PXD) technique was applied using a special designed cell^51^. This cell with sapphire capillary allows performing measurements under controlled gas atmosphere in a pressure range from 0.01 to 200 bar. Measurements were performed at Deutsches Elektronen-Synchrotron (DESY) in the P02.1 beamline. The beamline is equipped with A Perkin Elmer XRD1621 area detector and 60 keV X-ray source (λ = 0.207 Å). Mg-Li-5LTOK sample was heated from room temperature (RT) to 300 °C with a heating rate of 5 °C/min under 1 bar of H_2_ pressure. Every 10 seconds a two-dimensional SR-PXD pattern was collected. Collected data were integrated to one-dimensional diffraction pattern using Fit2D software^52,53^.

Differential scanning calorimetry (DSC) measurements were performed in a Netsch DSC 204 HP calorimeter located inside an argon-filled glovebox (H_2_O and O_2_ levels below 1 ppm). Before starting the DSC measurements, the residual argon gas inside the chamber was removed by first evacuation and then flushing the chamber with hydrogen. A mass flow-meter was used to limit the deviation of the hydrogen pressure to ±0.2 bar of H_2_ during heating up and cooling down. In order to measure apparent activation energies of the 1^st^ and 2^nd^ desorption, about 10–15 mg of each sample were placed in a Al_2_O_3_ crucible and then heated from room temperature (RT) up to 300 °C under 1 bar of H_2_ pressure with heating rates of 1 °C, 3 °C, 5 °C and 10 °C/min. For the 2^nd^ desorption, as-milled samples were first desorbed by heating from RT to 220 °C under 1 bar of H_2_ pressure. Following this step, the materials were reabsorbed by heating them from RT to 180 °C under 100 bar of H_2_ pressure.

In order to evaluate the effectiveness of the additives on the material kinetic behaviour, the apparent activation energies (*E*_*a*_) of the 1^st^ and 2^nd^ desorption reactions were calculated *via* Kissinger method^54^. This method is suitable for the samples that exhibit multi-step reactions and it allows us to determine *E*_*a*_ of a reaction process without assuming a specific kinetic model, i.e. without determining the rate-limiting step of the reaction. The equation for the *E*_*a*_ calculation is shown in Eq. 3;

3 $$ln(\beta /{T}_{m}^{2})=ln(AR/{E}_{a})-\frac{{E}_{a}}{R{T}_{m}},$$

where *A* is the pre-exponential factor and *R* is the gas constant. The temperature for the maximum reaction rate (T_m_) was obtained from DSC curves measured at measured heating rates (β) of 1 °C, 3 °C, 5 °C and 10 °C/min. Then, $ln(\frac{\beta }{{T}_{m}^{2}})$ against 1/T_m_ was plotted, *E*_*a*_ (kJ/mol H_2_) and *A* (1/s) was calculated from linear fitting. Goodness of fit was determined by the examining the correlation between the experimental and predicted values. In order to have a good fitting, R-square value should be near 1^50^.

In order to assess the rate-limiting steps of the absorption/desorption processes in the studied system, Sharp and Jones method was applied^55,56^. In this method, experimental data are expressed as following:

4 $$F(\alpha )=A(\frac{t}{{t}_{0.5}}),$$

where A is the rate constant, *t*_0_._5_ is the time at the reaction fraction α = 0.5. The fraction (α) is taken as the hydrogen capacity over the maximum reached capacity for each sample. By implementing different rate equations, several plots of ${(\frac{t}{{t}_{0.5}})}_{{\rm{theoretical}}}$ versus ${(\frac{t}{{t}_{0.5}})}_{{\rm{experimental}}}$ are obtained. In this study, we applied this model to the 1^st^, 2^nd^ and 5^th^ absorption/desorption curves between 0.1 and 0.8 fractions of the overall hydrogen capacity. The best fitting reaction rate model must obey the following rules; slope of the fitted line should be ∼1, intercept ∼0 and *R*^2^ ∼ 1. Details related to the implemented rate equations are given in our previous work^50^.

IR spectroscopy was performed with an Agilent Technologies Cary 630 FT-IR located in an argon filled glove box (H_2_O and O_2_ levels below 1 ppm). Each measurement was acquired in the transmission mode in spectral range of 650 cm^−1^–4000 cm^−1^ with a resolution of 4 cm^−1^ ^50^.

Evolved gases during the desorption reactions were analysed using a Hiden Analytical HAL 201 Mass-Spectrometer, which is coupled with a Netzsch STA 409 C Differential Thermal Analysis (DTA-MS). About 2 mg of sample was placed in a Al_2_O_3_ crucible that was heated from room temperature up to 300 °C in the DTA apparatus, with a heating rate of 3 °C/min. Measurements were done under 50 ml/min Ar flow.

The absorption rates and gravimetric capacities were assessed using a Sieverts apparatus (HERA Hydrogen Storage Systems, Longueil, QC, Canada) operating on the differential pressure technique. The hydrogen gas used in the experiments had a purity of 99.999 % (5.0 H_2_). The temperature and pressure conditions are provided in the figure caption for each experiment in the manuscript. The mass of sample for all the measurements was approximately 100 mg.

High resolution transmission electron microscopy (HR-TEM) observations, diffraction patterns (DP) and dark field (DF) were carried out using a Tecnai G2 microscope with an information limit of 0.12 nm and Schottky Emission gun operating at 300 kV. Samples after milling and after absorption/desorption conditions were observed. All samples were prepared into a glove box with controlled O_2_ and H_2_O atmosphere (<1 ppm) by dispersing the powders onto carbon grids. In order to avoid the oxidation/hydrolysis of the material at the time to introduce the grids into the microscope column, the dispersed powder on the grid was covered with a special polymeric film which does not preclude the electron interactions with the sample^57^. Then, HR-TEM observations of the identified Fe zones were done. HR-TEM image processing was done with the following programs: Digital Micrograph (License no. 90294175), i-TEM (License no. A2382500, EMSIS GmbH, Münster, Germany).

X-ray absorption spectroscopy experiments at the XANES (X-ray absorption near edge structure) region of LiTi_2_O_4_, K-modified additive (10 wt.% K_1_._04_O_16_Ti_8_, 17 wt.% LiTi_2_O_4_, 27 wt.% LiTiO_2_ and 46 wt.% K_2_O_17_Ti_8_), TiO_2_ anatase, as as-milled Mg-Li-5LTO, as-milled Mg-Li-5LTOK, as-milled Mg-Li-2.5LTOK, desorbed Mg-Li-2.5LTOK and reabsorbed Mg-Li-2.5LTOK samples were carried out using a R-XAS looper “in house” spectrometer from Rigaku. The measurements were performed in transmission mode around the Ti K-edge (4966 eV) in the range of energy from 4950 eV to 5030 eV at ambient temperature. The optimum amount of material for the measurements was calculated by the program XAFSMAS (version 2012/04, ALBA synchrotron, Barcelona, Spain)^58^. The samples were prepared inside a glove box by mixing them with anhydrous boron nitride (powder, purity: 98 %; Sigma-Aldrich, St. Louis, Missouri, MO, USA,) in a mortar, and then pressing the mixture into pellets of 10 mm diameter. The pellets were sealed with Kapton tape (50 μm in thickness) to prevent the oxidation/hydrolysis of the samples. XAS data processing and fitting were performed by using the IFEFFIT software (version 1.2.11, University of Chicago, Chicago, IL, USA) package^59^.

## Results

Results obtained from the thermal behaviour, mass spectroscopy, desorption activation energy, volumetric measurements, *ex situ* PXD, *in situ* SR-PXD and infrared spectroscopy for all the compositions are presented in this section. In Table 1, the starting stoichiometric compositions of all additives were shown and details regarding the additive synthesis were discussed in the experimental section. Rietveld refinement result of the PXD data for the 0.5LiH + TiO_2_ stoichiometric composition indicates that the additive is just composed of the LiTi_2_O_4_ after milling and annealing (ESI Fig. S1). In the case of the 0.5LiH + TiO_2_ + 0.25KH stoichiometric composition, after the synthesis the additive is composed of 10 wt.% K_1_._04_O_16_Ti_8_, 17 wt.% of LiTi_2_O_4_, 27 wt.% LiTiO_2_ and 46 wt.% K_2_O_17_Ti_8_ (ESI Fig. S2).

### First desorption/absorption performance and apparent activation energies

**Figure 1:**
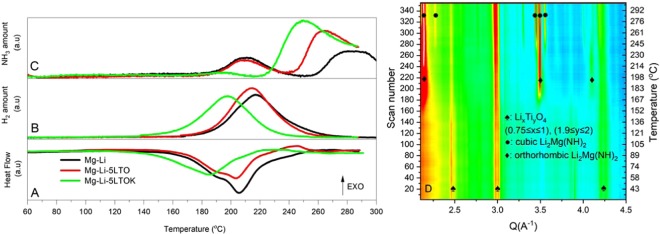
(**A**) DTA, (**B**,**C**) MS traces of as-milled samples measured in the temperature range of 60 °C–300 °C with a heating ramp of 3 °C/min and 50 ml/min Ar flow. (**D**) *In situ* SR-PXD data of the Mg-Li-5LTOK sample which was heated from RT to 300 °C with a heating ramp of 5 °C/min under 1 bar of Ar pressure.

The thermal behaviour of as-prepared samples is presented in Fig. 1A. The DTA curve of the additive-free sample Mg-Li exhibits two endothermic events between 170 °C and 230 °C. These two events are due to desorption reactions in accordance with Eqs. 1 and 2. Temperature of the desorption peak maximum is at 205 °C. Mg-Li-5LTO sample shows a desorption trend similar to that of Mg-Li. The presence of the additive does not lead to improvement neither the onset nor the peak maximum temperatures. MS analyses of the gases (H_2_ and NH_3_) evolving from the two samples upon heat treatment are almost identical (Fig. 1B,C). However, the sample containing K-modified additive, Mg-Li-5LTOK, shows a reduction of 30 °C on desorption onset temperature and the peak maximum of the main thermal event. Moreover, the release of NH_3_ is suppressed until 220 °C. Similar positive effects of K-based additives on the amide-hydride systems were reported previously in the literature^40^. In order to understand the processes taking place upon desorption, evolution of the crystalline phases were studied by *in-situ* SR-PXD (Fig. 1D). The PXD pattern acquired at RT reveals that the reflections are ascribable to the presence of the additive (LiTi_2_O_4_). However, due to the broadness of the observed diffraction peaks, we cannot exclude the presence of several phases having a general formula Li_x_Ti_y_O_4_ (0.75 ≤ x ≤ 1, 1.9 ≤ y ≤ 2). This fact suggests that the additive´s composition changes upon milling. The presence of reflections belonging to Li_2_Mg(NH)_2_ (orthorhombic phase) at around 170 °C indicates that the desorption reaction has already started, which is in good agreement with DTA analysis (Fig. 1A). The formation of the cubic Li_2_Mg(NH)_2_ takes place at the temperatures higher than 220 °C. This transition is expected since the phase transformation of Li_2_MgN(H)_2_ from the orthorhombic to the cubic structure occurs over 200 °C^60^. Unfortunately, in this analysis, it was not possible to identify any crystalline potassium compounds. This implies that potassium-containing phases are either in amorphous or nanocrystalline state.

**Figure 2:**
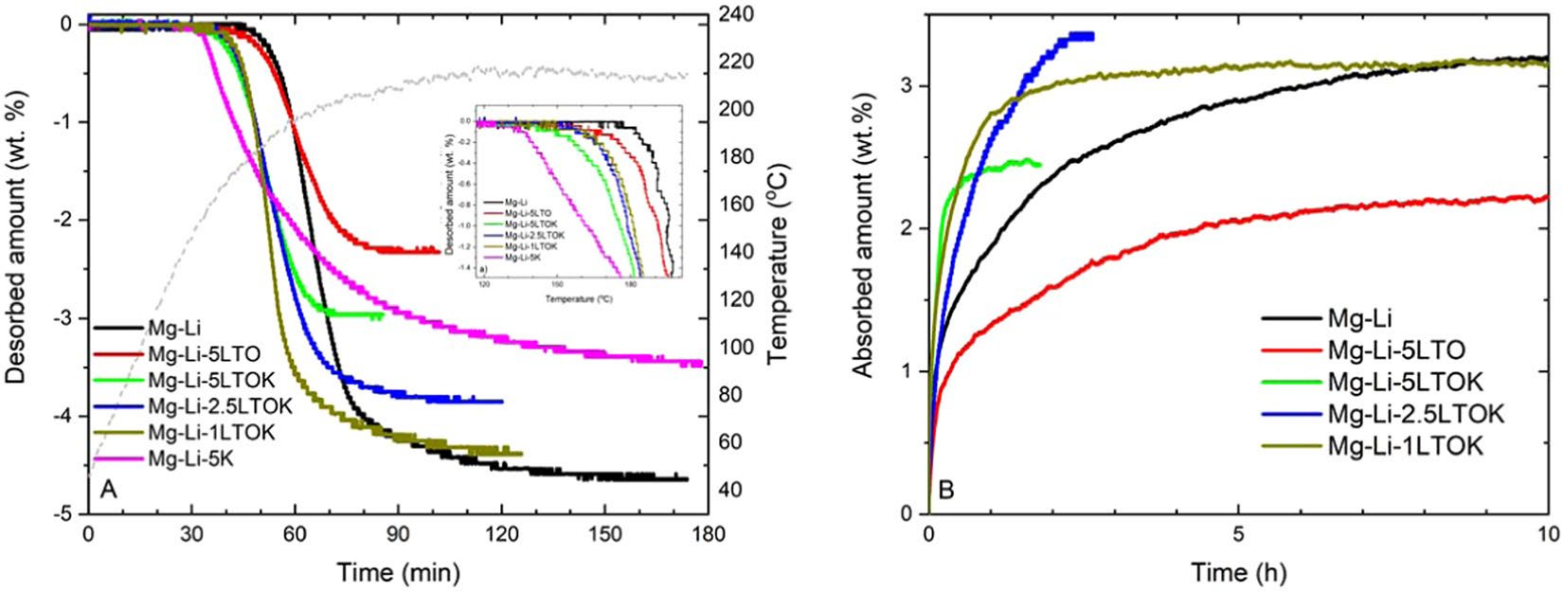
(**A**) 1^st^ Desorption kinetics of as-milled samples (RT→ 220 °C under 1 bar of H_2_, 3 °C/min heating rate) (**B**) Reabsorption kinetics at 180 °C (isothermal) and 80 bar of H_2_ pressure.

First desorption kinetics of as-milled samples are presented in Fig. 2A. Desorption of Mg-Li starts at 180 °C and 4.5 wt.% of gas is released within 120 minutes. Mg-Li-5LTO displays a similar behaviour as Mg-Li, though with a reduced capacity to 2.3 wt.% due to the presence of significant amount of additive (26 wt.%). Modifying the additive with potassium (Mg-Li-5LTOK, Mg-Li-2.5LTOK and Mg-Li-1LTOK) leads to a notable reduction on desorption onset temperature from 180 °C to 150 °C. This temperature reduction to some extent changes with the amount of LTOK additive. It is possible to observe in the inset plot of Fig. 2A that higher additive amounts lead to slightly lower onset temperatures. Additionally, DSC analyses shows that the onset temperature of Mg-Li-2.5LTOK is about 15 °C lower than that of Mg-Li-1LTOK (ESI Fig. S3). Clearly, the decrement of the amount of LTOK lead to an increase in the desorbed gas amount. As seen in Fig. 2A, Mg-Li-5LTOK desorbs 3 wt.%, whereas Mg-Li-2.5LTOK and Mg-Li-1LTOK desorbs 3.8 wt.% and 4.3 wt.%, respectively. K-containing additives, especially KH, are known to improve reaction kinetics of Mg(NH_2_)_2_ + LiH system^40–42^. In order to compare our findings with the pure KH added system, Mg-Li-5K sample (Mg(NH_2_)_2_ + 2LiH + 0.05KH) was prepared. Despite the fact that the lowest onset temperature (135 °C) is obtained with this sample, its reaction rate is slower in comparison with the samples containing K-modified additive.

Reabsorption kinetics of Mg-Li and Mg-Li-5LTO are sluggish and require more than 10 hours to reach full capacity (Fig. 2B). On the contrary, K-modified samples absorb H_2_ notably faster (Mg-Li-5LTOK within 1 hour, Mg-Li-2.5LTOK within 2.5 hours and Mg-Li-1LTOK within 2 hours). Therefore, the effect of K-modified additive on Mg-Li is clearly seen both in the absorption and desorption kinetic properties. Despite the fact that Mg-Li-1LTOK has fast H_2_ absorption kinetic, the H_2_ capacity is reduced from 4.3 to 3 wt. % after a single cycle. After cycling, the best sample that has a good H_2_ absorption kinetic and cycling stability is Mg-Li-2.5LTOK. For this reason, we chose this sample to further investigate its cycling stability compared to Mg(NH_2_)_2_ + 2LiH system.

**Figure 3:**
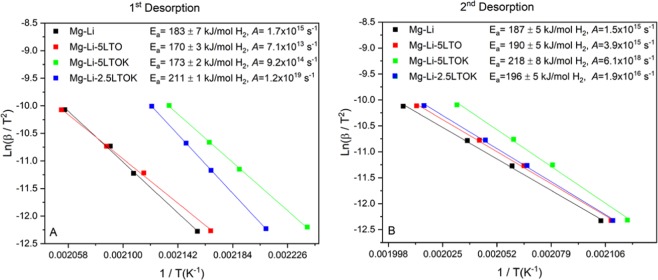
Kissinger plots of samples: (**A**) 1^st^ desorption, (**B**) 2^nd^ desorption derived from DSC curves at different heating rates (1, 3, 5 and 10 °C/min) for the calculation of the *E*_*a*_.

In order to evaluate the effect of the modified additives on desorption apparent activation energy (*E*_*a*_), the Kissinger method^54^ was applied for the 1^st^ and 2^nd^ desorption reactions of the Mg-Li, Mg-Li-5LTO, Mg-Li-5LTOK and Mg-Li-2.5LTOK samples. For the calculations, the peak maximum of the main thermal event (ESI: Figs. S4–S7) was considered for the calculations of the *E*_*a*_. Figure 3A,B show the Kissinger plots and values of *E*_*a*_. It is possible to see that the 1^st^ desorption reaction of Mg-Li has an activation energy of 183 ± 7 kJ/mol H_2_ (Fig. 3A). The presence of the additives 5LTO and 5LTOK lowers *E*_*a*_ down to 170 ± 3 kJ/mol H_2_ and 173 ± 2 kJ/mol H_2_ respectively, as well as the frequency factor (*A*). On the contrary, *E*_*a*_ value rises to 211 ± 1 kJ/mol H_2_ for the sample Mg-Li-2.5LTOK. It is worthy to note that frequency factor of this sample is considerably higher (*A* = 1.2 × 10^19^ s^−1^) compared to the ones of the Mg-Li, Mg-Li-5LTO and Mg-Li-5LTOK samples.

The *E*_*a*_ values calculated for the 2^nd^ desorption reactions (Fig. 3B) increase in comparison with the 1^st^ desorption, except for Mg-Li-2.5LTOK, which decreases by nearly 15 kJ/mol. It is worthy to note that the experiments were repeated in order to confirm this trend. Taking into account the error bands (ESI – Fig. S7), the *E*_*a*_ values for Mg-Li, Mg-Li-5LTO and Mg-Li-2.5LTOK overlap. However, the frequency factor for Mg-Li-2.5LTOK is higher than the ones for Mg-Li and Mg-Li-5LTO. The highest values of *E*_*a*_ and *A* were measured for Mg-Li-5LTOK. It is also noticed that K-containing additives reduce the desorption peak temperature both in the 1^st^ and 2^nd^ desorption.

### Cycling stability

**Figure 4:**
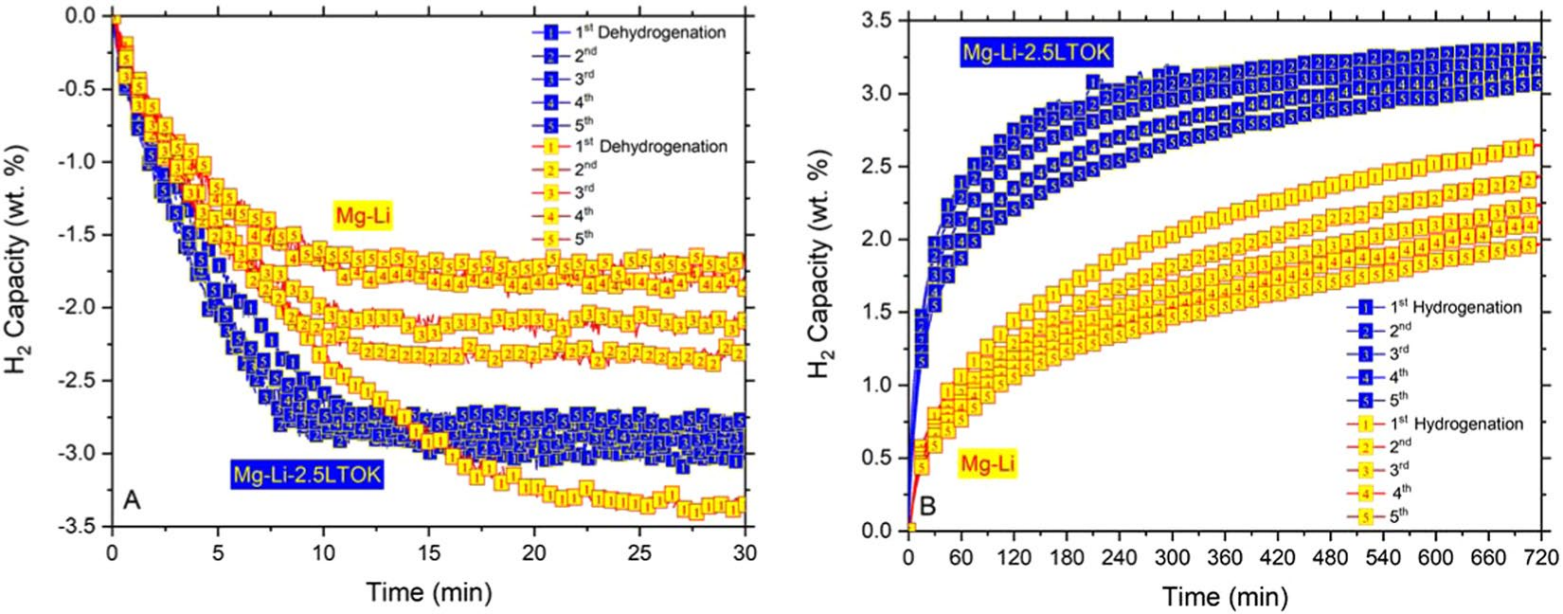
Reaction kinetics of Mg-Li and Mg-Li-2.5LTOK within the first 5 H_2_ absorption/desorption cycles, (**A**) Isothermal desorption at 180 °C and 1 bar of H_2_ pressure (**B**) Isothermal absorption at 180 °C and 80 bar of H_2_.

In Figs. 1 and 2, it was shown that LTOK additive improves the hydrogen storage properties of the Mg(NH_2_)_2_ + 2LiH hydride system, i.e. reduced desorption temperature, fast reabsorption kinetic. Mg-Li-2.5LTOK sample exhibited the highest reversible H_2_ storage capacity of about 3.5 wt. % (Fig. 2). Hence, this subsection presents its cycling stability/reversibility in comparison with the sample without additive, i.e. Mg-Li. Figure 4 shows the cycling stability upon 5 absorption/desorption processes for Mg-Li and Mg-Li-2.5LTOK samples. During cycling, both desorption and absorption kinetics of Mg-Li-2.5LTOK are 2 and 5 times faster, respectively, than those of Mg-Li. In addition, the hydrogen storage capacity of Mg-Li is reduced by a half after 5 cycles, from 3.4 to 1.7 wt. %, whereas the cycling process reduces only in 10% the hydrogen capacity, i.e. from 3.1 to 2.75 wt. %, in the case of Mg-Li-2.5LTOK sample. From Fig. 4B, it is observed that measurement time of 12 hours is not enough for the complete absorption in Mg-Li sample, whereas Mg-Li-2.5LTOK reaches almost equilibrium at this time.

### Initial structural analysis

**Figure 5:**
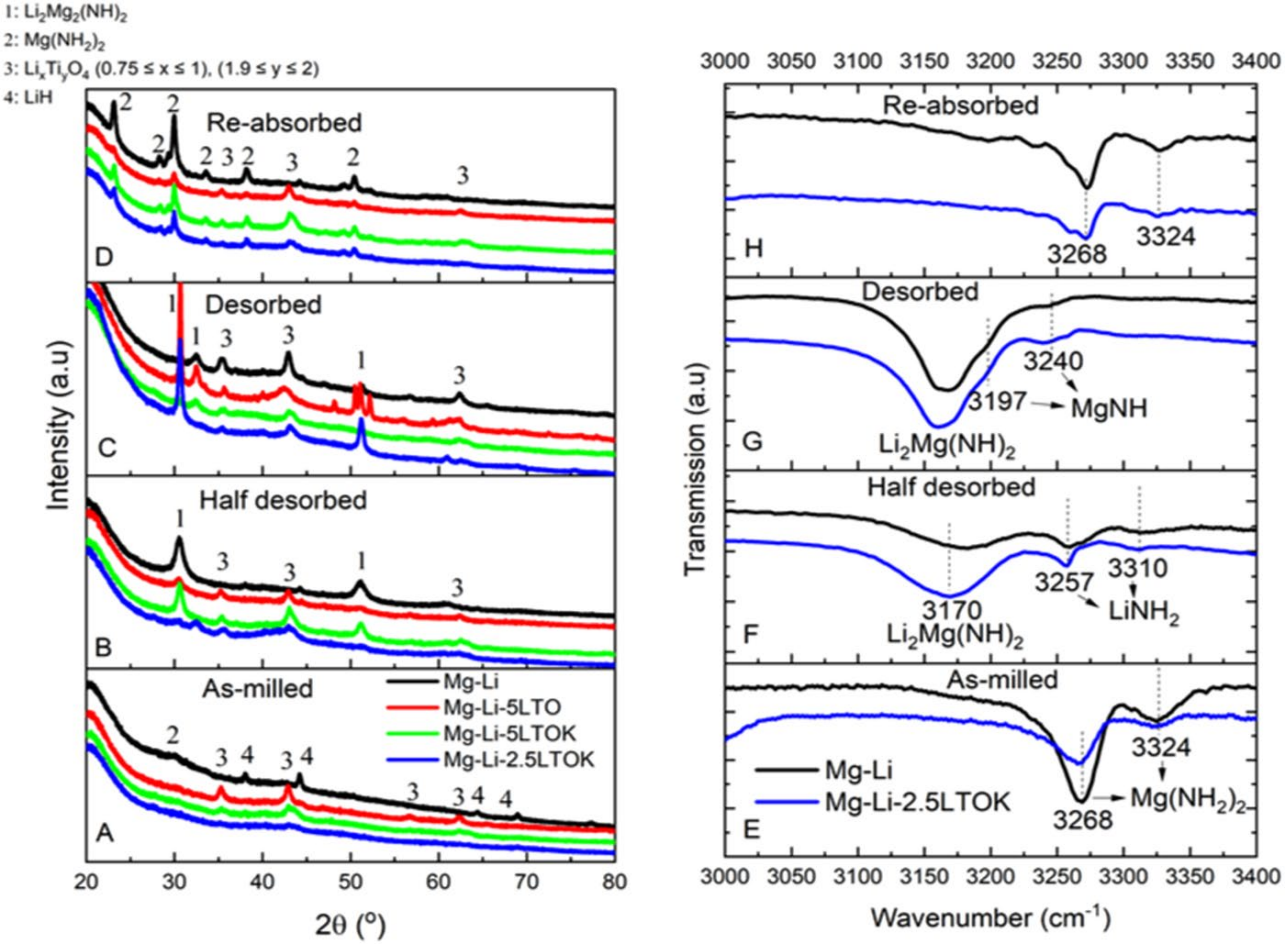
(**A**–**D)** PXD plots of samples at different reaction states. (**E**–**H)** Corresponding FT-IR plots of samples Mg-Li and Mg-Li-2.5LTOK. Desorption and absorption were performed under 1 bar of H_2_ at 210 °C and 80 bar of H_2_ pressure at 180 °C, respectively.

First overview to the structural analysis was done with PXD and FT-IR techniques (Fig. 5A–D,E–H, respectively). PXD patterns of the samples after ball milling (Fig. 5A) exhibit broad peaks with low intensity, which can be attributed to the harsh milling conditions. As-milled Mg-Li contains cubic LiH structure with *Fm*3*m(225)* space group and a broad peak at 2θ = 30°, which corresponds to the tetragonal Mg(NH_2_)_2_ structure with *I*4_1_*/acd(142)* space group. Since Mg(NH_2_)_2_ is amorphous after intense ball milling, it can be hardly observed in PXD^61^. In contrast, it is more visible on the FT-IR pattern (Fig. 5E), where N-H stretching vibrations of Mg(NH_2_)_2_ are positioned at 3268 and 3324 cm^−1^. When Mg-Li is half desorbed, LiNH_2_ can be detected at 3257 and 3310 cm^−1^ (Fig. 5F). Fully desorbed sample contains small bumps at 3240 and 3197 cm^−1^, which correspond to IR signals from MgNH (Fig. 5G)^62^. LiNH_2_ and MgNH products from the desorption of the sample should have a solid-solid reaction to form a ternary imide: Li_2_Mg(NH)_2_^63^. PXD reflections coming from the cubic Li_2_Mg(NH)_2_ phase with *iba*2*(45)* space group are found in the half and fully desorbed samples (Fig. 5B,C). This imide is also observed by FT-IR at 3170 cm^−1^ (Fig. 5F,G). Absorption of the desorbed Mg-Li at 180 °C leads to recrystallization of Mg(NH_2_)_2_ (Fig. 5D).

Regarding the additives, PXD analyses (Fig. 5A–D) reveal that in all cases Li_x_Ti_y_O_4_ compounds with 0.75 ≤ x ≤ 1 and 1.9 ≤ y ≤ 2 are present. In the ICSD database, it is possible to find several crystal structures belonging to Li_x_Ti_y_O_4_ that fit well with all reflections^64^. These formed phases are stable and their peaks positions do not change within desorption/absorption processes. The compositions of the as-synthesized additives were already presented in the introduction of the results section (ESI Figs. S1 and S2). However, it is observed that further mechanical milling of these additives with Mg(NH_2_)_2_ and LiH leads to some changes in the additives´ composition, which will be later discussed in the following section.

## Discussion

In this work, microstructural and kinetic effects of Li_x_Ti_y_O_z_ and K-modified Li_x_Ti_y_O_z_ additives on the Mg(NH_2_)_2_ + 2LiH system were studied. K-modified additive not only plays a role on improving the reaction kinetic behaviour (Fig. 2) and cycling stabilities (Fig. 4), but also helps lowering the desorption onset and peak temperatures (Fig. 1A) in comparison to the pristine sample (Mg-Li). Mg-Li releases NH_3_ at the cycling temperature of 180 °C, which is comparably lower respect to the release of H_2_. However, the suppression of NH_3_ release at this temperature was achieved by the addition of LTOK (Fig. 1C). Then, the H_2_ storage capacity was optimized by tuning the amount of additive. Thus, a reversible H_2_ capacity of about 3 wt. % at 180 °C was achieved for Mg-Li-2.5LTOK upon cycling (Fig. 4). FT-IR analyses carried out for the sample Mg-Li and Mg-Li-2.5LTOK after milling, after desorption and absorption (Fig. 5E–H) confirmed that the reaction pathway described in reactions (1) and (2), section 1, is not altered.

**Figure 6:**
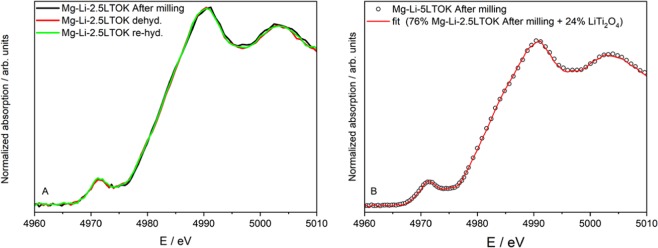
(**A**) XANES spectra at the Ti K-edge of Mg-Li-2.5LTOK after milling (black line), after dehydrogenation (red line) and after rehydrogenation (green line). (**B**) Linear combination fit (red line) for the XANES spectrum of Mg-Li-5LTOK (circles).

As we reported in Fig. 5, the composition of the additive after milling with Mg(NH_2_)_2_ and LiH changes. XRD analyses of the as-milled materials (Fig. 5A,D) provided a hint about the presence of stable Li_x_Ti_y_O_4_ compounds (0.75 ≤ x ≤ 1 and 1.9 ≤ y ≤ 2). Nevertheless, the composition of the additives in the LTOK after milling is not clear yet. Therefore, X-ray absorption spectroscopy near edge structure (XANES) technique was applied to Mg-Li-5LTO, Mg-Li-5LTOK and Mg-Li-2.5LTOK samples in order to investigate the oxidation state of Ti. The changes in the oxidation state of Ti were determined by the shift of the absorption edges of the samples. The results were compared with the measured XANES spectra of TiO_2_ and LiTi_2_O_4_ reference materials. In Fig. 6A, the spectra of the Mg-Li-2.5LTOK after milling, desorption and reabsorption are compared. It is possible to observe that all spectra are similar, thus the nature of the LTOK additive does not change upon hydrogen interaction. It was found that the oxidation state of Ti in both additives LTO (LiTi_2_O_4_: 100 %, ESI Fig. S1) and LTOK (10 wt.% K_1_._04_O_16_Ti_8_, 17 wt.% of LiTi_2_O_4_, 27 wt.% LiTiO_2_ and 46 wt.% K_2_O_17_Ti_8_, ESI Fig. S2) is the same (ESI Fig. S8). Durmeyer *et al*. already reported that Ti in LiTi_2_O_4_ has an effective valence state of +3.5^65^. Thus, the effective oxidation state of Ti in LTO and LTOK additives is +3.5. Comparing the XANES spectra of the as-milled Mg-Li-5LTO, the LTO additive and anatase TiO_2_ (ESI Fig. S9A), it is possible to observe a change in the position of the absorption edge towards higher energies for the Mg-Li-5LTO respect to LTO additive. Hence, this indicates that the valence state of Ti atoms in Mg-Li-5LTO is, on average, higher than +3.5 and lower than +4. A similar behaviour is observed for the Mg-Li-5LTOK sample (ESI Fig. S9A), with a slightly shift toward higher energies on the absorption edge respect to Mg-Li-5LTO. This fact suggests that a different titanium compound could be formed in the potassium-containing samples. If we compare two samples with different LTOK additive loads (Mg-Li-5LTOK and Mg-Li-2.5LTOK), the absorption edge of both samples seems to be similar, showing that the average Ti valence in this samples is very close (ESI Fig. S9B). Then, the results from the Fig. S9 show that the effective valence state of the Ti atoms in the samples slightly depends on the presence of the K-based additive. Based on the analysis above, it is possible to reproduce the Mg-Li-5LTOK spectrum with 76 % of Mg-Li-2.5LTOK and 24 % of LTO additive (LiTi_2_O_4_) as shown in Fig. 6B. Thus, K-modified additive in the Mg-Li-5LTOK sample is composed of 24 % of LiTi_2_O_4_ (Ti^+3^.^5^) and 76 % of other species, suggesting that the effective Ti valence state is slightly smaller than the presented by the Mg-Li-2.5LTOK sample.

**Figure 7:**
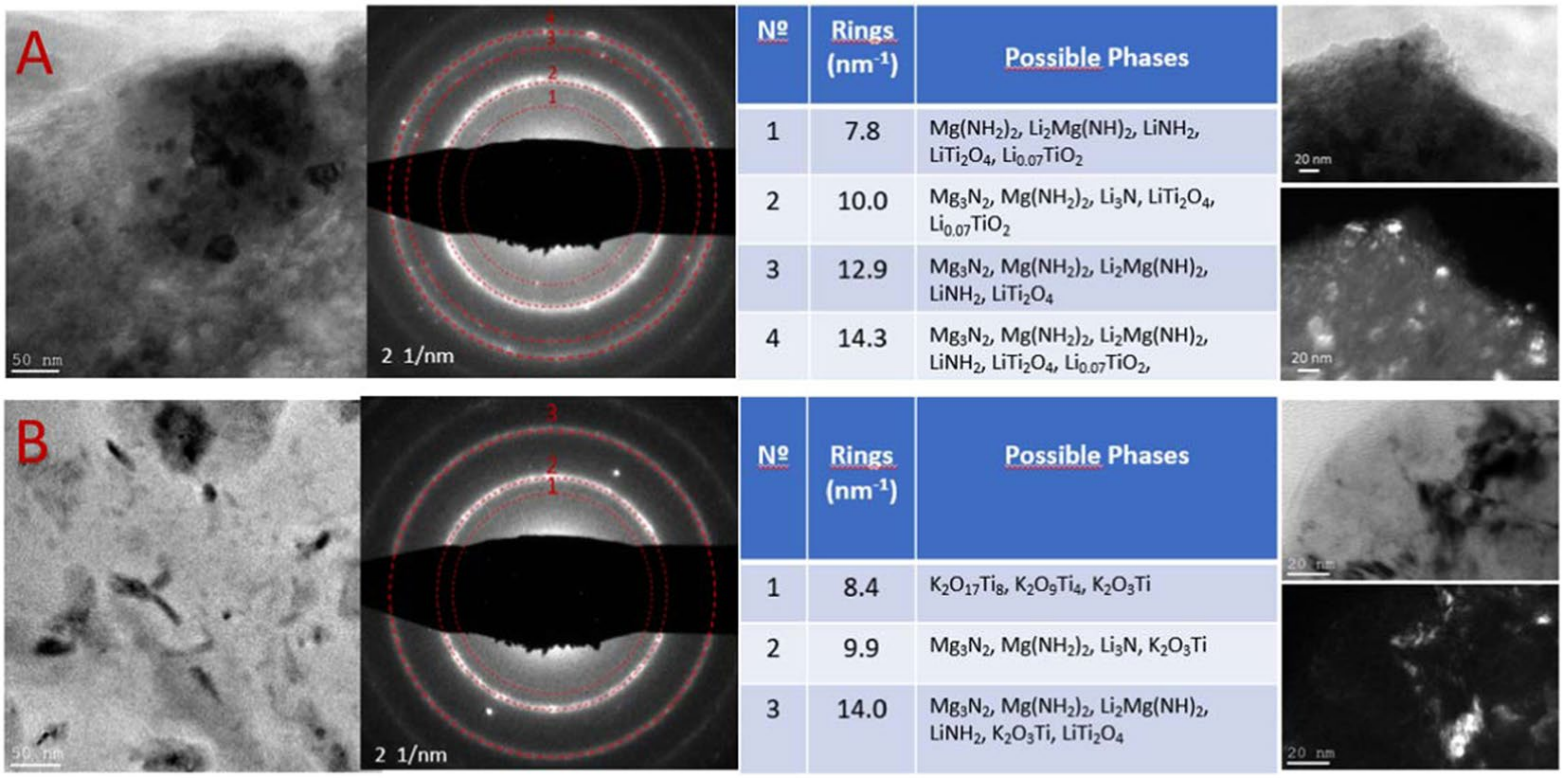
TEM bright field photos (BF, left), diffraction patterns (DP, middle), table for possible phases and, BF+DF set of images (right column): (**A**) Mg-Li-5LTO and (**B**) Mg-Li-2.5LTOK.

TEM observations and analyses were performed to determine the nature of the formed additives upon milling. Figure 7 shows bright field TEM photos (BF), diffraction patterns (DP) and tables of possible phases based on the DP and dark field images (DF), for the as-milled Mg-Li-5LTO and Mg-Li-2.5LTOK samples. The DP of as-milled samples were taken in the region showed by the BF images. Reflections from the DP are related to the main phase of the material, Mg(NH_2_)_2_ compound. Due to the thickness of the rings, it is not possible to discard some intermediate (LiNH_2_), product (Li_2_Mg(NH)_2_) and by-product (Li_3_N and Mg_3_N_2_) species. However, these phases are not expected in the as-milled state, unless Mg(NH_2_)_2_ and LiH interacts during the observations prompted by the energy of the beam. It is also possible to attribute the observed reflections to species composed of Li-Ti-O (Fig. 7A) and K-Ti-O (Fig. 7B). On the one hand, species such as LiTi_2_O_4_ and Li_0_._07_TiO_2_ are found for the Mg-Li-5LTO sample. On the other hand, species composed of K-Ti-O as well as LiTi_2_O_4_ are present in the Mg-Li-2.5LTOK sample. Dark field images formed from 3^rd^ and 4^th^ rings for Mg-Li-5LTO and from the 3^rd^ ring for Mg-Li-2.5LTOK shows small nanoparticles in the range of 5 to 30 nm that can be attributed to the Li-Ti-O and K-Ti-O species.

**Figure 8:**
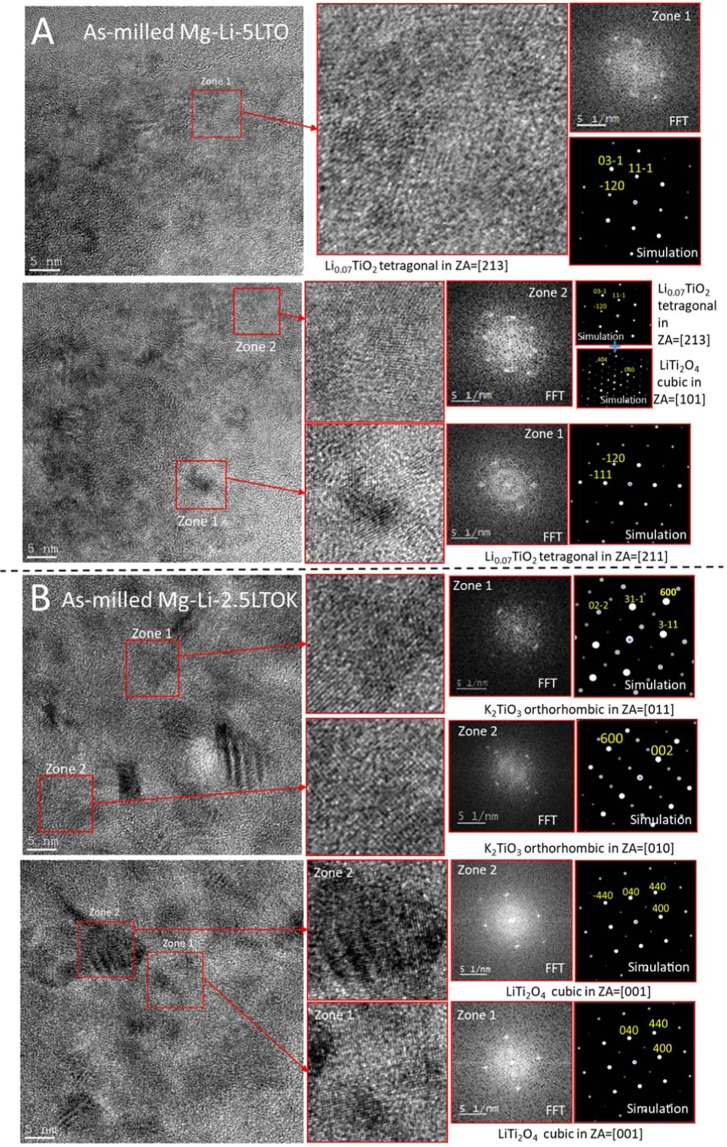
Characterization of the nano-sized Li-Ti-O and K-Ti-O for (**A**) as-milled Mg-Li-5LTO and (**B**) as-milled Mg-Li-2.5LTOK by means of HR-TEM. FFT was calculated in each region and compared to simulated diffraction patterns (DPs) in the adequate orientation.

In order to verify the formation of such Li-Ti-O and K-Ti-O nanoparticles, HR-TEM observation, fast Fourier transform (FFT) and crystal structure simulation analyses were performed. Figure 8 shows the HR-TEM of the as-milled Mg-Li-5LTO and as-milled Mg-Li-2.5LTOK along with its FFTs calculated in each region, and compared to simulated diffraction patterns (DPs). In the as-milled Mg-Li-5LTO (Fig. 8A), the presence of nanoparticles of Li_0_._07_TiO_2_ (tetragonal) and LiTi_2_O_4_ (cubic) are confirmed by the structure analyses of the HR-TEM photos. For the as-milled Mg-Li-2.5LTOK (Fig. 8B), nanoparticles of K_2_TiO_3_ (orthorhombic) and LiTi_2_O_4_ (cubic) are found. Based on the position of the absorption edge of the Mg-Li-2.5LTOK sample compared to the ones from references TiO_2_ and LiTi_2_O_4_ (Fig. S9B), we can attribute those titanium atoms in the sample has an average valence state higher than +3.5 and close to +4.

**Figure 9:**
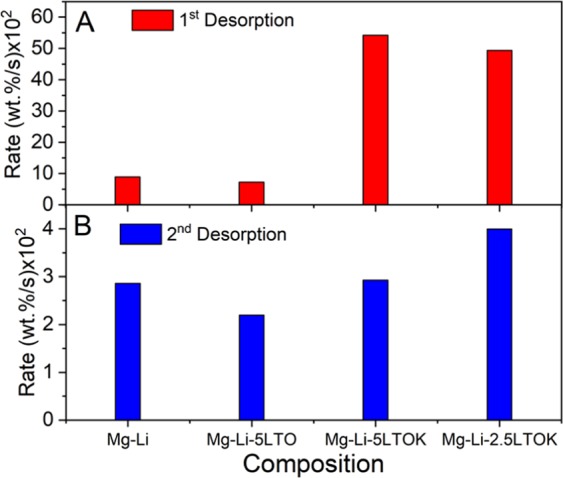
Reaction rate for the first and second desorption processes for the investigated samples.

In terms of the observed improved kinetic behaviour (Fig. 2), and the calculated desorption *E*_*a*_ for the first and second desorption reactions (Fig. 3), we can find some unexpected results. On one hand, the Mg-Li-2.5LTOK sample clearly shows reduced onset temperature upon the first desorption and faster kinetic during the first (Fig. 2A), second and subsequent absorption/desorption cycles (Fig. 4) in comparison with the Mg-Li sample. Moreover, among the samples with additives, the Mg-Li-2.5LTOK sample exhibit higher capacity (~3 wt.%) and faster absorption kinetics (Fig. 4). On the other hand, the activation energy values are higher than the one of the material without additive, Mg-Li (Fig. 3). In order to shed light onto this fact, the kinetic constant (*k*) was calculated by the Arrhenius expression *k* = A · exp[−*E*_*a*_/RT] (1/s) at 180 °C, which is the cycling temperature (Fig. 4). Then, to take into account the effect of the capacity of each sample, *k* was multiplied by the capacity after reabsorption taken from Fig. 2B, which can be considered as the more realistic value (ESI – Table S1). As seen in Fig. 9, the desorption rate upon the first and second desorption reactions for the samples with the addition of LTOK is faster than the ones for the Mg-Li and Mg-Li-5LTO samples. However, the activation energies for LTOK containing samples are similar or higher. This behaviour can be mainly attributed to an increase in the frequency factor, making possible a more efficient contacting of the reactants on the interphase.

The faster rate of the sample with 5-mol. % LTOK during the first desorption is in agreement with the lower *E*_*a*_ in comparison with sample with 2.5-mol. % LTOK, suggesting a better distribution of the additive. However, during the second desorption, the beneficial effects of the larger amount of additive is lost, hinting that the additive might have agglomerated and then acting as a barrier for the reactants interactions. Moreover, adding 5-mol. % LTOK leads to a notable drop in the desorbed gas amount.

In order to further investigate the role of the additive on the system, an analysis on the rate-limiting steps^66^ of Mg-Li and Mg-Li-2.5LTOK samples is carried out for the 1^st^,2^nd^ and 5^th^ absorption/desorption kinetic curves from Fig. 4 (ESI Figs. S10–S21; Tables S2–S6). The results are summarized in Table 2. Desorption rates are limited by an interface controlled mechanism (F1: JMA, n = 1), while absorption rates are limited by a diffusion controlled mechanism. In the case of absorption reaction, D3 and D4 represent diffusion mechanisms as rate limiting step, but with different geometries of praticles (D3: spheres and D4: different forms). Therefore, K-modified additive does not change the rate-limiting step for desorption/absorption reactions. However, in both absorption and desorption mechanisms, the rate-limiting step is notably accelerated. In general, the results are in well agreement with the results obtained from our previous work, where amide/hydride molar ratio was 6/9 instead of 1/2^50^.

**Table 2:** Using Sharp and Jones method^55,56^, rate-limiting processes of samples, which were taken from isothermal cycling kinetic curves of Fig. 4.

Desorption	
1^st^ Desorption	F1
2^nd^ Desorption	F1
5^th^ Desorption	F1
**Absorption**	
1^st^ Absorption	D3
2^nd^ Absorption	D4
5^th^ Absorption	D3

F1 : JMA, n = 1, Random nucleation, one-dimensional interface controlled growth.

D3 : Three-dimensional diffusion, spherical particles.

D4 : Three-dimensional diffusion, free geometry.

Therefore, these outcomes suggest that the presence of the K_2_TiO_3_ species account for the observed improvements in the kinetic behaviour and cycling stability of the Mg-Li-2.5LTOK. Kinetic enhancements from the alkali metals and their hydrides/hydroxides/amides are still discussed, whether they modify thermodynamics of the system or they have a catalytic effect on the system^32,37,38,41,67–70^. Catalytic activity of KH and RbH was explained *via* destabilization of N-H bond due to their high electronegativity^32^. KH firstly reacts with Mg(NH_2_)_2_ and later metathesizes with LiH to regenerate KH^41^. Based on our results from *in situ* SR-PXD contour plot (Fig. 1D) together with XANES spectra (Fig. 6B), we propose that K_2_TiO_3_ does not take part in the reactions, but its presence can positively affect the reversible reactions of the Mg(NH_2_)_2_ + 2LiH system due to high electronegativity of K (0.82 eV), Ti (1.54 eV) and O (3.44 eV) elements. Therefore its acts as a catalyst rather than changing the thermodynamics of the system.

## Conclusions

In this work, microstructural and kinetic effects of Li_x_Ti_y_O_z_ and K-modified Li_x_Ti_y_O_z_ additives on the Mg(NH_2_)_2_ + 2LiH system were studied. 5 mol. % additive containing sample Mg-Li-5LTOK reduced the desorption peak temperature of pristine sample by 30 °C and suppressed NH_3_ release until 220 °C. Although Mg-Li-2.5LTOK has comparably higher apparent activation energy (211 ± 1 kJ/mol) respect to Mg-Li (183 ± 7 kJ/mol), calculated rate constant (*k*) value was bigger during the first and second desorption reactions which is in agreement with reaction behaviour. Orthorhombic K_2_TiO_3_ and cubic LiTi_2_O_4_ phases were detected in HR-TEM observations, where oxidation state of Ti was in accordance with XANES analysis. Based on our results from *in situ* SR-PXD plot and XANES analysis, we propose that K_2_TiO_3_ nanoparticles act as catalyst and they positively affect the reversible reactions of the Mg(NH_2_)_2_ + 2LiH system due to high electronegativity of K (0.82 eV), Ti (1.54 eV) and O (3.44 eV) elements.

## Supplementary information

Improved kinetic behavior of Mg(NH2)2-2LiH doped with nanostructured K-modified-LixTiyOz for hydrogen storage

## Data Availability

The datasets generated during and/or analysed during the current study are available from the corresponding authors on reasonable request.
