# Supplementary material for: Improved kinetic behaviour of Mg(NH2)2-2LiH doped with nanostructured K-modified-LixTiyOz for hydrogen storage
Source: Sci Rep. 2020 Jan 7;10:8. doi: 10.1038/s41598-019-55770-y (PMC6946654; doi:10.1038/s41598-019-55770-y)
Supplement: Supplementary file 1 — Improved kinetic behavior of Mg(NH2)2-2LiH doped with nanostructured K-modified-LixTiyOz for hydrogen storage [file 41598_2019_55770_MOESM1_ESM.pdf]

Improved kinetic behavior of $Mg(NH_2)_2$-2LiH doped with nanostructured K-modified-$Li_xTi_yO_z$ for hydrogen storage

Gökhan Gizer[1*], Julián Puszkiel[1,2], Maria Victoria Castro Riglos[1,2], Claudio Pistidda[1**], José Martín Ramallo-López[3], Martin Mizrahi[3], Antonio Santoru[1], Thomas Gemming[4], Jo-Chi Tseng[5], Thomas Klassen[1] and Martin Dornheim[1***]

[1] Institute of Materials Research, Materials Technology, Helmholtz-Zentrum Geesthacht GmbH, Max-Planck Strasse 1, D-21502 Geesthacht, Germany

[2] Consejo Nacional de Investigaciones Científicas y Técnicas (CONICET), Centro Atómico Bariloche, Av. Bustillo km 9500, S.C. de Bariloche, Argentina

[3] Instituto de Investigaciones Fisicoquímicas Teóricas y Aplicadas, INIFTA (CCT La Plata-CONICET, UNLP), Diagonal 113 y Calle 64, 1900 La Plata, Argentina

[4] IFW Dresden, P.O. Box 270016, D-01171 Dresden, Germany

[5] Deutsches elektronen-Synchrotron, Notkestr. 85, 22607 Hamburg

Corresponding Authors: *Gökhan Gizer (goekhan.gizer@hzg.de), **Claudio Pistidda (claudio.pistidda@hzg.de), ***Martin Dornheim (martin.dornheim@hzg.de)

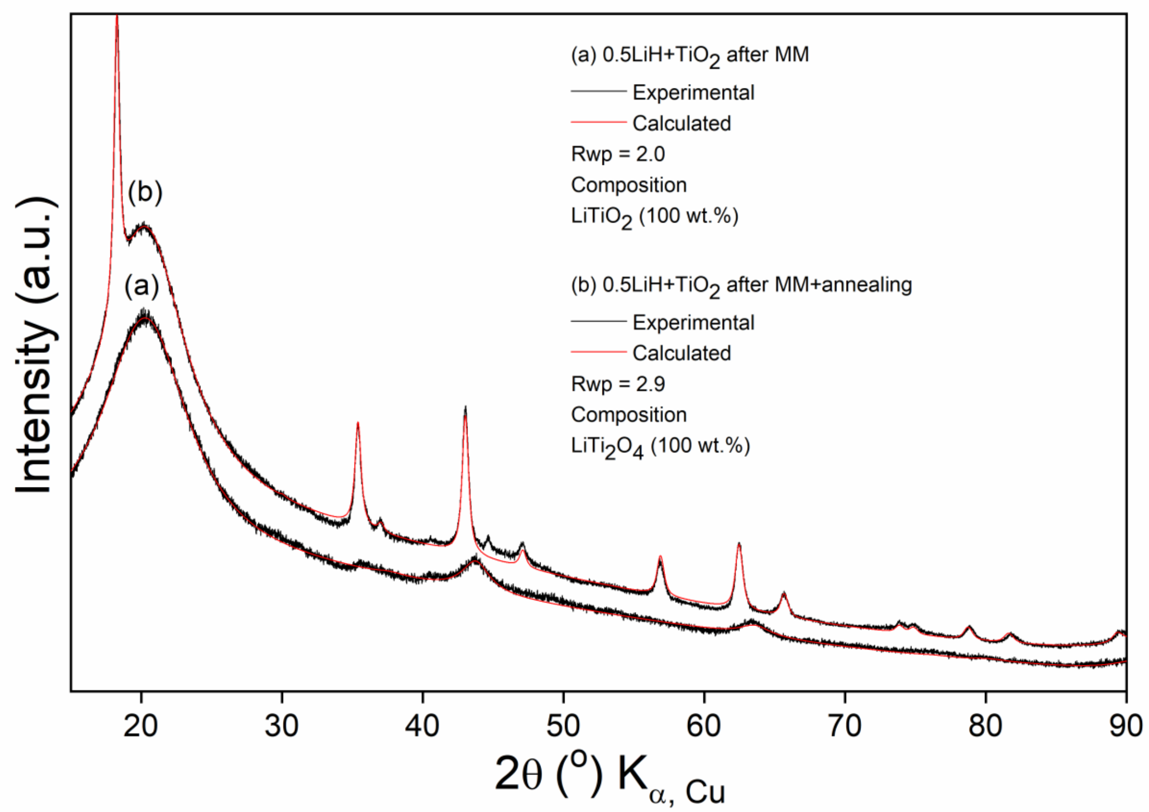

Figure S1 PXD of 0.5LiH+TiO_2 **(a)** after milling, **(b)** after milling and annealing

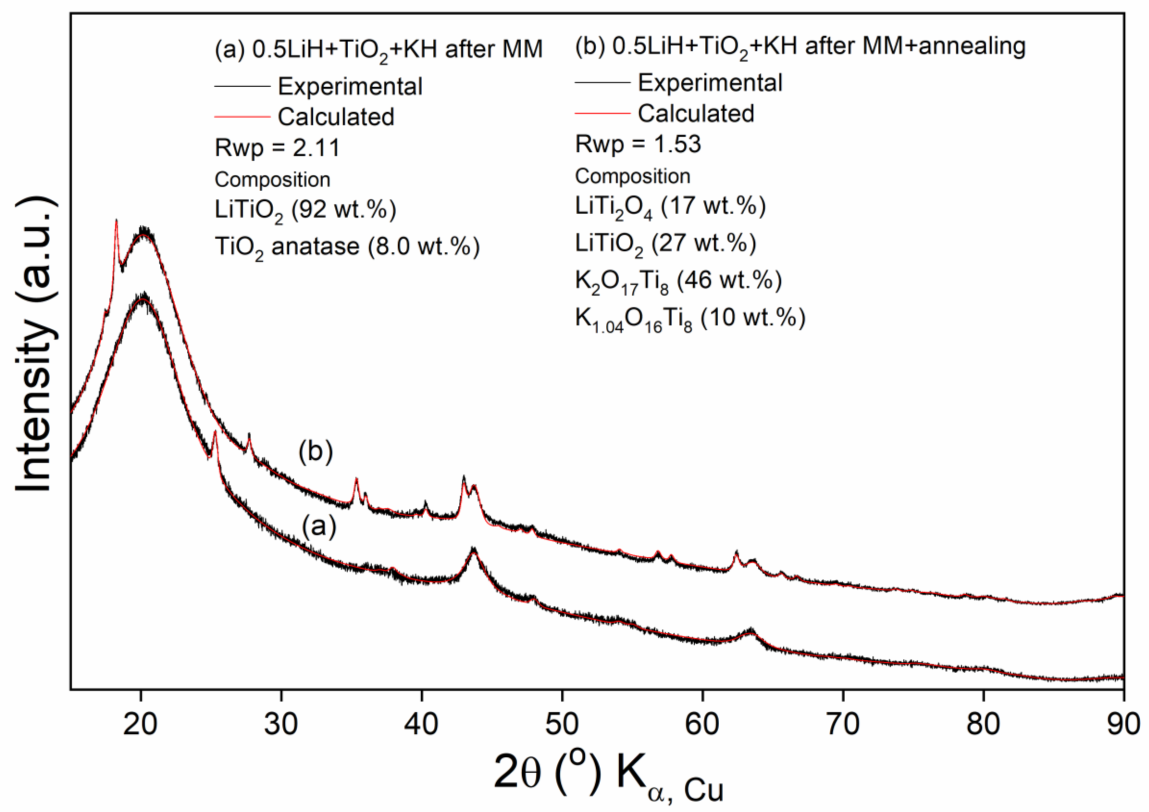

Figure S2 PXD of 0.5LiH+TiO_2+0.25KH, **(a)** after milling **(b)** after milling and annealing

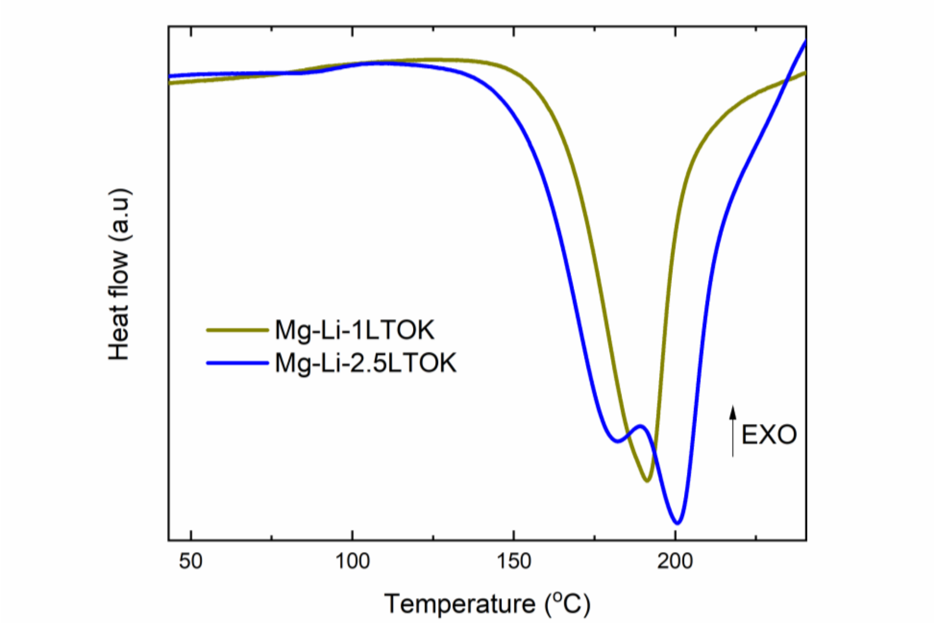

Figure S3 DSC curves of Mg-Li-2.5LTOK and Mg-Li-1LTOK

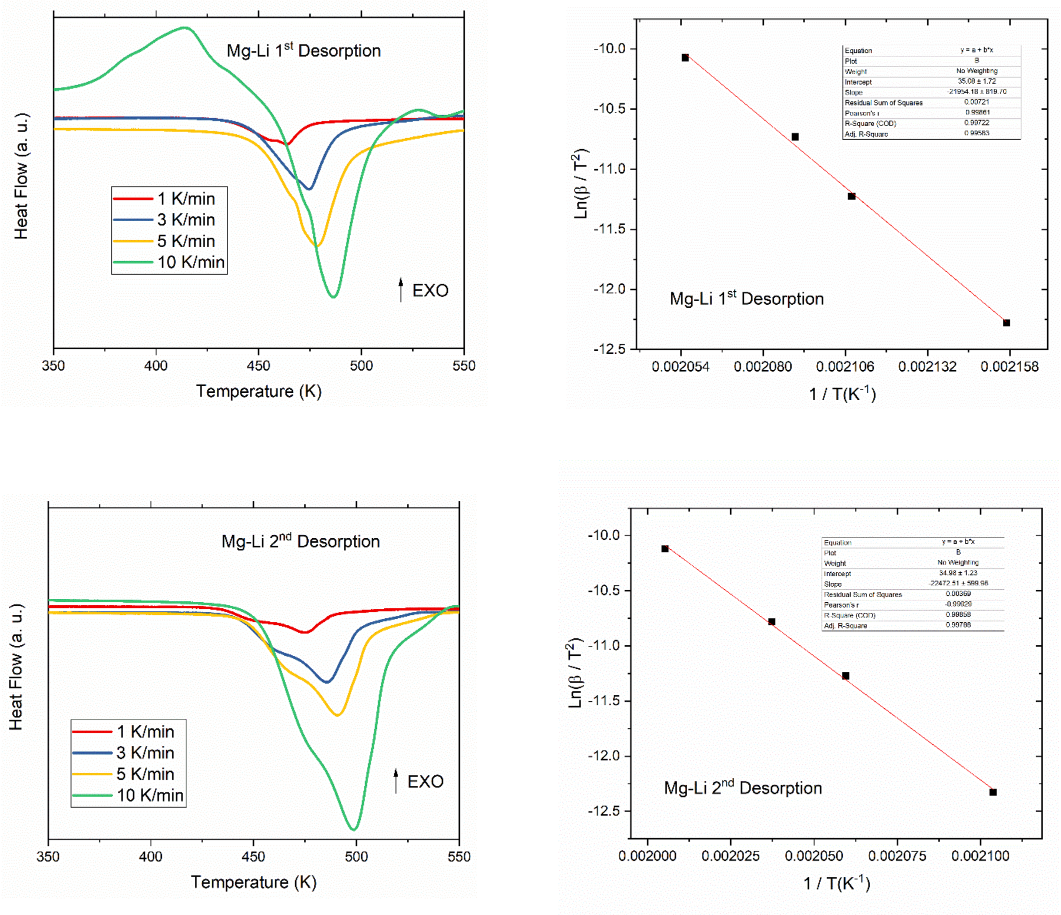

Figure S4 DSC and corresponding Arrhenius plots of Mg-Li at the 1st and 2nd desorption

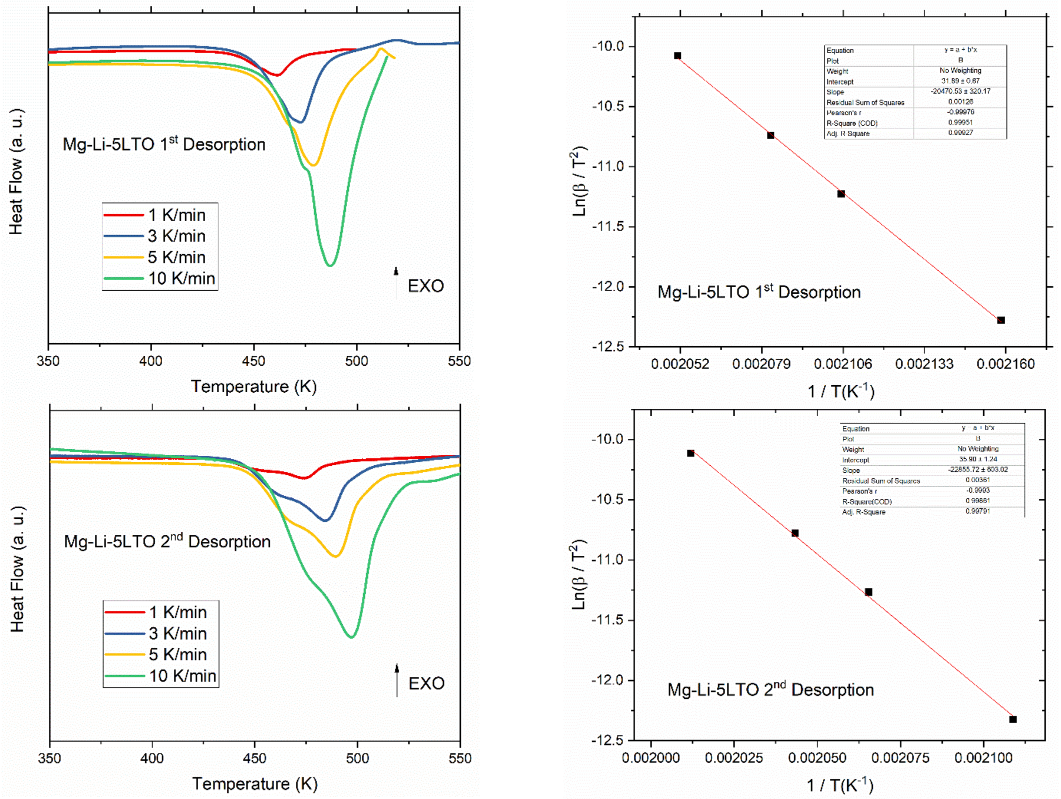

Figure S5 DSC and corresponding Arrhenius plots of Mg-Li-5LTO at the 1st and 2nd desorption

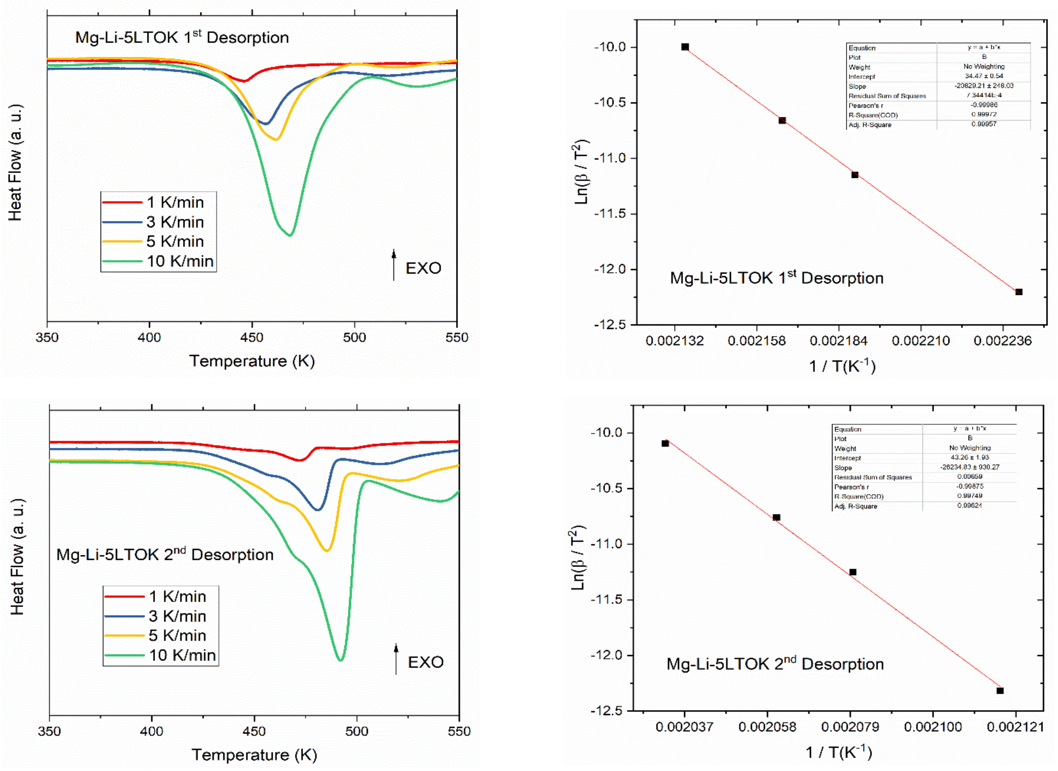

Figure S6 DSC and corresponding Arrhenius plots of Mg-Li-5LTOK at the 1st and 2nd desorption

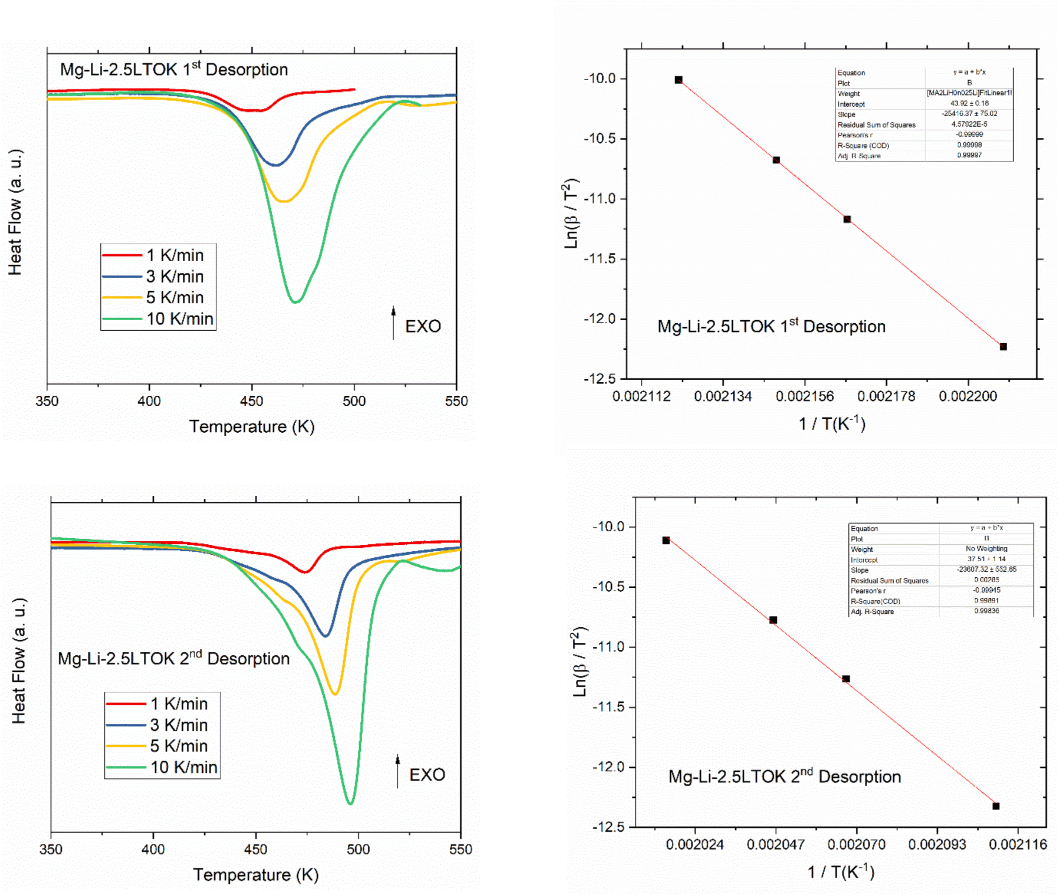

Figure S7 DSC and corresponding Arrhenius plots of Mg-Li-2.5LTOK at the 1st and 2nd desorption

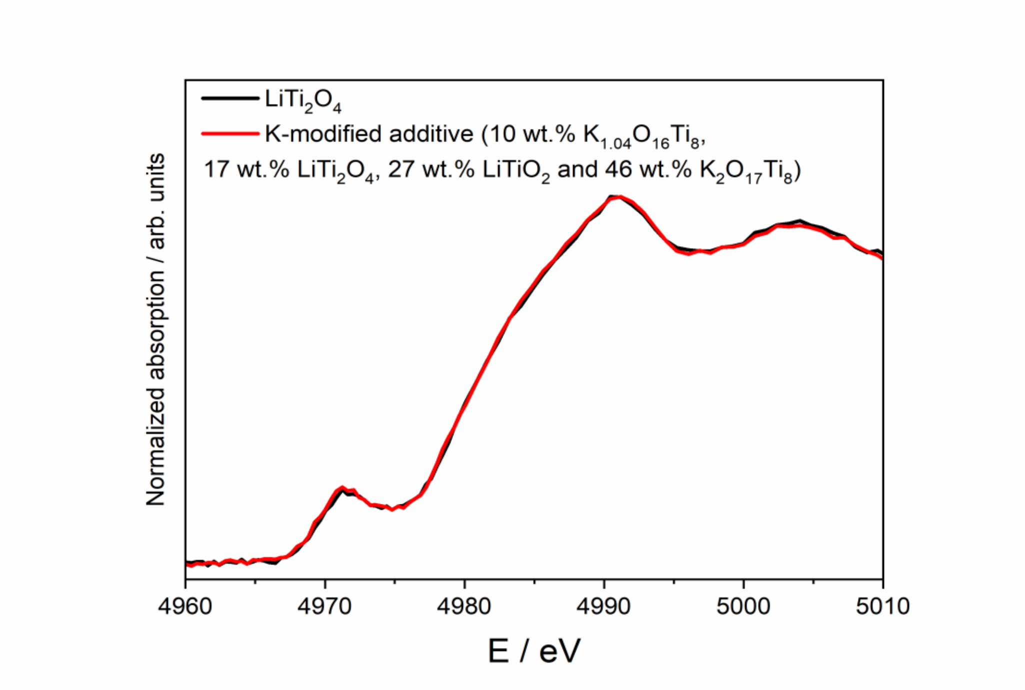

Figure S8 XANES spectra at the Ti K-edge of $LiTi_2O_4$ (black line) and K-modified additive (red line).

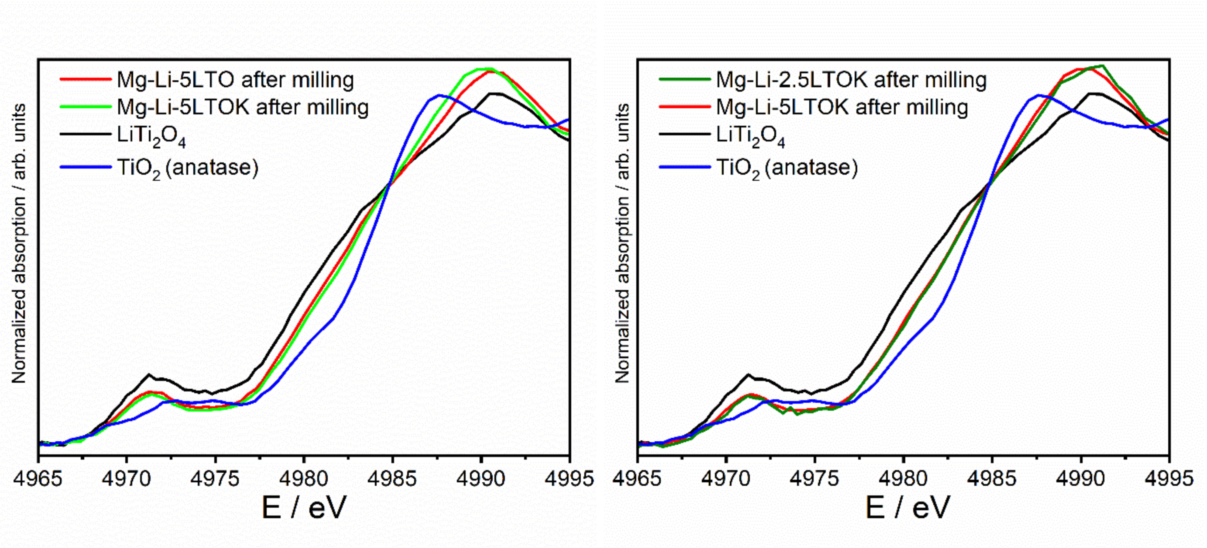

Figure S9 XANES curves for: **A** Mg-Li-5LTO and Mg-Li-5LOTK and **B** Mg-Li-2.5LTOK and Mg-Li-5LTOK. $LiTi_2O_4$ and TiO_2 (anatase) are the references.

Table S1 Calculation of the kinetic constant applying the Arrhenius equation

Composition	A (1/s)	Ea1 (kJ/mol)	1st Dehydrogenation / k = A * exp [-Ea/RT]x10^2	H2 capacity x k (wt.%/s)
Mg-Li	3.77E+19	183±7	3.0	8.9
Mg-Li-5LTO	1.45E+18	170±3	3.6	7.2
Mg-Li-5LTOK	1.93E+19	173±2	21.7	54.3
Mg-Li-2.5LTOK	3.02E+23	211±1	14.1	49.3
**Composition**	**A (1/s)**	**Ea2 (kJ/mol)**	**2nd Dehydrogenation / k = A * exp [-Ea/RT]x10^2**	**H2 capacity x k (wt.%/s)**
Mg-Li	3.49E+19	187±5	1.0	2.9
Mg-Li-5LTO	8.92E+19	190±5	1.1	2.2
Mg-Li-5LTOK	1.61E+23	218±8	1.2	2.9
Mg-Li-2.5LTOK	4.56E+20	196±5	1.1	4.0

**Table S2 −** Fitted rate equations	
**Kinetic rate models**	**Rate equations to be used for Sharp and Jones Method**
D1 one-dimensional diffusion	$\frac{\alpha^2}{0.25}$
D2 two-dimensional diffusion	$\frac{\alpha + ((1-\alpha) * \ln(1-\alpha))}{0.1534}$
D3 Jander eq. for three dimensional diffusion	$\frac{(1-(1-\alpha)^{\frac{1}{3}})^2}{0.04255}$
D4 Ginstling-Braunshtein eq. for three dimensional diffusion	$\frac{1-\frac{2}{3}\alpha-(1-\alpha)^{\frac{2}{3}}}{0.0367}$
F1 JMA - n = 1	$\frac{-\ln(1-\alpha)}{0.6931}$
R2 two - dimensional interface controlled	$\frac{1-(1-\alpha)^{\frac{1}{2}}}{0.29289}$
R3 three dimensional interface controlled	$\frac{1-(1-\alpha)^{\frac{1}{3}}}{0.20629}$
F2 JMA - n = 1/2	$\frac{-\ln(1-\alpha)^{\frac{1}{2}}}{0.832}$
F3 JMA - n = 1/3	$\frac{-\ln(1-\alpha)^{\frac{1}{3}}}{0.8849}$
F4 JMA - n = 1/4	$\frac{-\ln(1-\alpha)^{\frac{1}{4}}}{0.9124}$
F5 JMA - n = 2/5	$\frac{-\ln(1-\alpha)^{\frac{2}{5}}}{0.8636}$

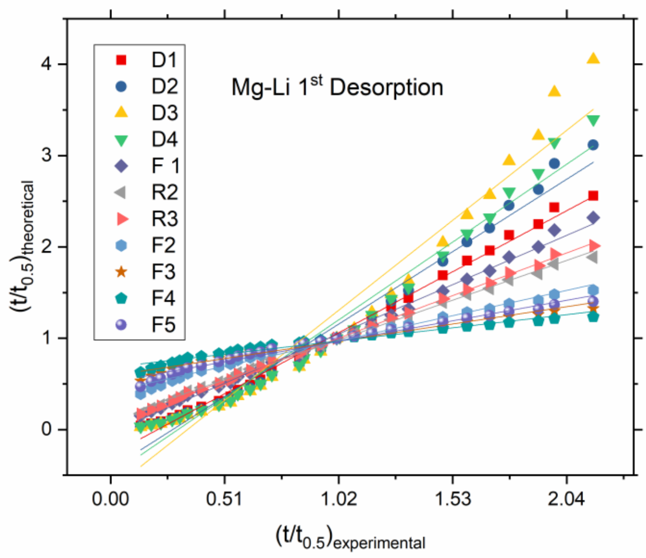

Figure S10 – $(t/t_{0.5})_{experimental}$ vs. $(t/t_{0.5})_{theoretical}$ plot for sample Mg-Li at 1st desorption

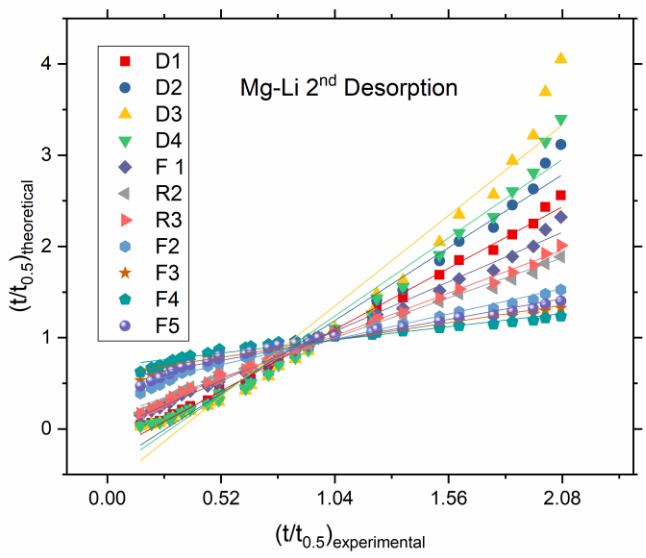

Figure S11 – $(t/t_{0.5})_{experimental}$ vs. $(t/t_{0.5})_{theoretical}$ plot for sample Mg-Li at 2nd desorption

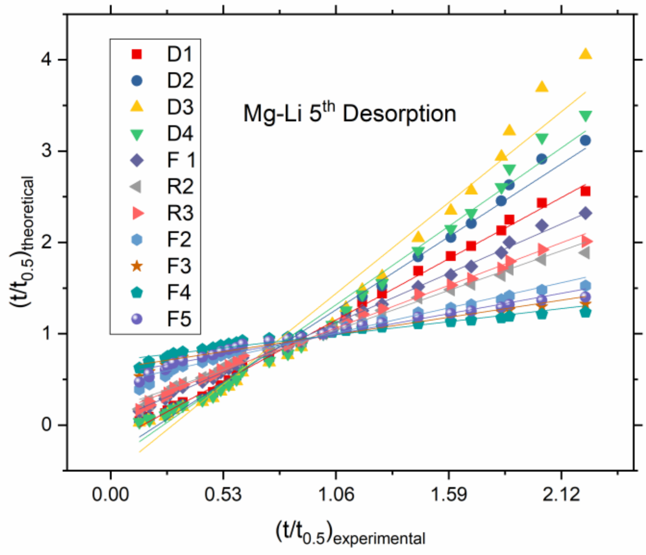

Figure S12 – $(t/t_{0.5})_{experimental}$ vs. $(t/t_{0.5})_{theoretical}$ plot for sample Mg-Li at 5th desorption

Table S3 Fitting results for the desorption curves of Mg-Li material.

$Mg(NH_2)_2$+2LiH (Mg-Li) 1st Desorption Fraction (0.10-0.80)	Intercept Value	Intercept Error	Slope Value	Slope Error	Statistics Adr. R-Square
D1 one-dimensional diffusion	-0.27626	0.02211	1.31016	0.01975	0.9939
D2 two-dimensional diffusion	-0.43203	0.04872	1.55547	0.04351	0.9793
D3 Jander eq. for three dimensional diffusion	-0.66457	0.09721	1.93167	0.08682	0.94818
D4 Ginstling-Braunshtein eq. for three dimensional diffusion	-0.50397	0.063	1.67114	0.05626	0.97027
F1 JMA - n = 1	-0.03271	0.01531	1.05852	0.01367	0.99552
R2 two - dimensional phase	0.11038	0.00959	0.85378	0.00856	0.99729
R3 three dimensional phase boundary	0.06666	0.00615	0.91517	0.00549	0.99903
F2 JMA - n = 1/2	0.41636	0.01364	0.54313	0.01218	0.98659
F3 JMA - n = 1/3	0.58797	0.01417	0.37134	0.01265	0.96958
F4 JMA - n = 1/4	0.68106	0.01275	0.2831	0.01138	0.9581
F5 JMA - n = 2/5	0.51712	0.01445	0.44051	0.01291	0.97732
$Mg(NH_2)_2$+2LiH (Mg-Li) 2nd Desorption Fraction (0.10-0.80)	Intercept Value	Intercept Error	Slope Value	Slope Error	Statistics Adr. R-Square
D1 one-dimensional diffusion	-0.25805	0.01988	1.29451	0.01773	0.99496
D2 two-dimensional diffusion	-0.41046	0.04688	1.53694	0.0418	0.9804
D3 Jander eq. for three dimensional diffusion	-0.63673	0.09623	1.90755	0.0858	0.9481
D4 Ginstling-Braunshtein eq. for three dimensional diffusion	-0.48057	0.06146	1.65099	0.0548	0.97108
F1 JMA - n = 1	-0.01609	0.01947	1.04386	0.01736	0.99259
R2 two - dimensional phase	0.12411	0.01464	0.84163	0.01306	0.99354
R3 three dimensional phase boundary	0.0812	0.01284	0.90231	0.01145	0.99567
F2 JMA - n = 1/2	0.42589	0.01662	0.53455	0.01482	0.97966
F3 JMA - n = 1/3	0.59482	0.0159	0.36512	0.01418	0.96083
F4 JMA - n = 1/4	0.68642	0.01399	0.27821	0.01248	0.94838
F5 JMA - n = 2/5	0.52508	0.01663	0.43331	0.01483	0.96932
$Mg(NH_2)_2$+2LiH (Mg-Li) 5th Desorption Fraction (0.10-0.80)	Intercept Value	Intercept Error	Slope Value	Slope Error	Statistics Adr. R-Square
D1 one-dimensional diffusion	-0.19098	0.01483	1.26539	0.01344	0.99696
D2 two-dimensional diffusion	-0.33515	0.03405	1.50708	0.03086	0.9888
D3 Jander eq. for three dimensional diffusion	-0.55048	0.07631	1.87837	0.06917	0.96463
D4 Ginstling-Braunshtein eq. for three dimensional diffusion	-0.40175	0.04625	1.62118	0.04193	0.98225
F1 JMA - n = 1	0.03675	0.01119	1.02174	0.01014	0.99735
R2 two - dimensional phase	0.16967	0.01733	0.82056	0.01571	0.9902
R3 three dimensional phase boundary	0.12904	0.01339	0.88082	0.01214	0.99489
F2 JMA - n = 1/2	0.45548	0.01819	0.52046	0.01649	0.97359
F3 JMA - n = 1/3	0.6156	0.01701	0.35488	0.01542	0.95141
F4 JMA - n = 1/4	0.70247	0.01481	0.27017	0.01342	0.93739
F5 JMA - n = 2/5	0.54947	0.01796	0.42145	0.01628	0.96121

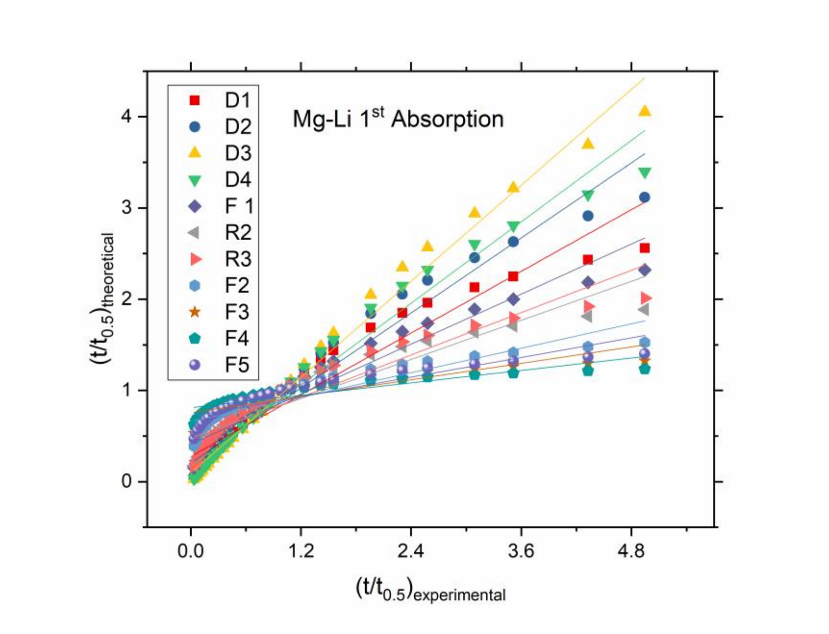

Figure S13 – $(t/t_{0.5})_{experimental}$ vs. $(t/t_{0.5})_{theoretical}$ plot for sample Mg-Li at 1st absorption

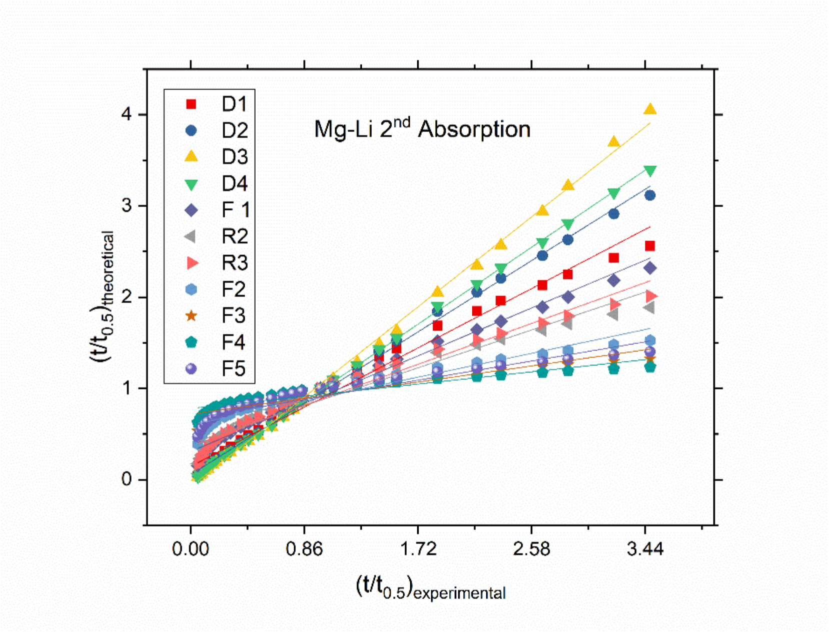

Figure S14 – $(t/t_{0.5})_{experimental}$ vs. $(t/t_{0.5})_{theoretical}$ plot for sample Mg-Li at 2nd absorption

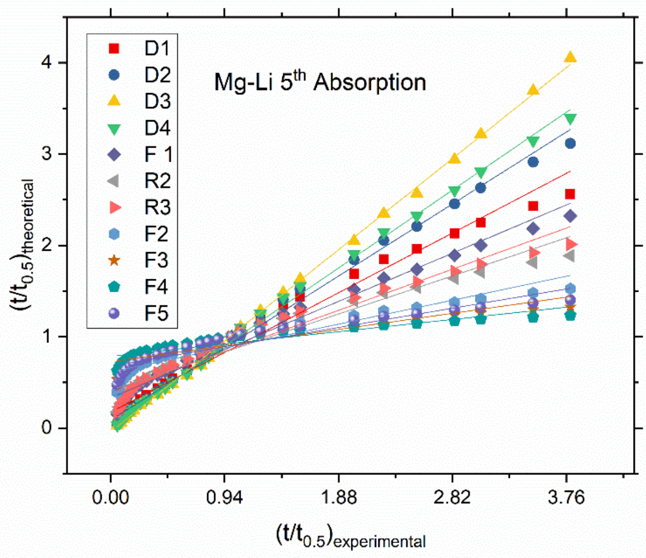

Figure S15 – $(t/t_{0.5})_{experimental}$ vs. $(t/t_{0.5})_{theoretical}$ plot for sample Mg-Li at 5th absorption

Table S4 Fitting results for the absorption curves of Mg-Li material.

$Mg(NH_2)_2$+2LiH (Mg-Li) 1st Absorption<br>Fraction (0.10-0.80)	Intercept Value	Intercept Error	Slope Value	Slope Error	Statistics Adr. R-Square
D1 one-dimensional diffusion	0.27581	0.05505	0.56387	0.03039	0.92707
D2 two-dimensional diffusion	0.20333	0.04848	0.68579	0.02676	0.96044
D3 Jander eq. for three dimensional diffusion	0.09406	0.03508	0.8764	0.01936	0.98698
D4 Ginstling-Braunshtein eq. for three dimensional diffusion	0.16967	0.04457	0.74409	0.0246	0.9713
F1 JMA - n = 1	0.41169	0.04221	0.4569	0.0233	0.93422
R2 two - dimensional phase	0.4829	0.04665	0.35708	0.02575	0.87629
R3 three dimensional phase boundary	0.46104	0.04556	0.38676	0.02515	0.89711
F2 JMA - n = 1/2	0.65776	0.0341	0.22355	0.01882	0.83836
F3 JMA - n = 1/3	0.75619	0.02648	0.15027	0.01462	0.79498
F4 JMA - n = 1/4	0.81051	0.02144	0.11357	0.01184	0.77131
F5 JMA - n = 2/5	0.71516	0.02988	0.17949	0.0165	0.81299
$Mg(NH_2)_2$+2LiH (Mg-Li) 2nd Absorption<br>Fraction (0.10-0.80)	Intercept Value	Intercept Error	Slope Value	Slope Error	Statistics Adr. R-Square
D1 one-dimensional diffusion	0.14562	0.02739	0.75576	0.01834	0.98435
D2 two-dimensional diffusion	0.05548	0.01268	0.90954	0.00849	0.99765
D3 Jander eq. for three dimensional diffusion	-0.07838	0.01611	1.14717	0.01079	0.99762
D4 Ginstling-Braunshtein eq. for three dimensional diffusion	0.01404	0.0057	0.98246	0.00382	0.99959
F1 JMA - n = 1	0.30895	0.02272	0.60986	0.01521	0.98346
R2 two - dimensional phase	0.39564	0.0323	0.48301	0.02163	0.94854
R3 three dimensional phase boundary	0.36899	0.02961	0.5209	0.01983	0.9623
F2 JMA - n = 1/2	0.60159	0.02632	0.30381	0.01762	0.91648
F3 JMA - n = 1/3	0.71707	0.02172	0.20546	0.01454	0.88036
F4 JMA - n = 1/4	0.78044	0.01803	0.15576	0.01207	0.85972
F5 JMA - n = 2/5	0.66909	0.02397	0.24481	0.01605	0.89565
$Mg(NH_2)_2$+2LiH (Mg-Li) 5th Absorption<br>Fraction (0.10-0.80)	Intercept Value	Intercept Error	Slope Value	Slope Error	Statistics Adr. R-Square
D1 one-dimensional diffusion	0.17338	0.03308	0.69585	0.0208	0.97642
D2 two-dimensional diffusion	0.08687	0.02004	0.8392	0.0126	0.99395
D3 Jander eq. for three dimensional diffusion	-0.04185	0.00957	1.06112	0.00602	0.99913
D4 Ginstling-Braunshtein eq. for three dimensional diffusion	0.04705	0.01341	0.90726	0.00843	0.99767
F1 JMA - n = 1	0.33098	0.02656	0.56183	0.0167	0.97668
R2 two - dimensional phase	0.41445	0.03524	0.44379	0.02216	0.93679
R3 three dimensional phase boundary	0.38879	0.03285	0.47902	0.02066	0.95211
F2 JMA - n = 1/2	0.61377	0.0279	0.27883	0.01755	0.90305
F3 JMA - n = 1/3	0.72558	0.02269	0.18833	0.01427	0.86509
F4 JMA - n = 1/4	0.787	0.01873	0.14267	0.01178	0.84364
F5 JMA - n = 2/5	0.6791	0.02518	0.22451	0.01583	0.88108

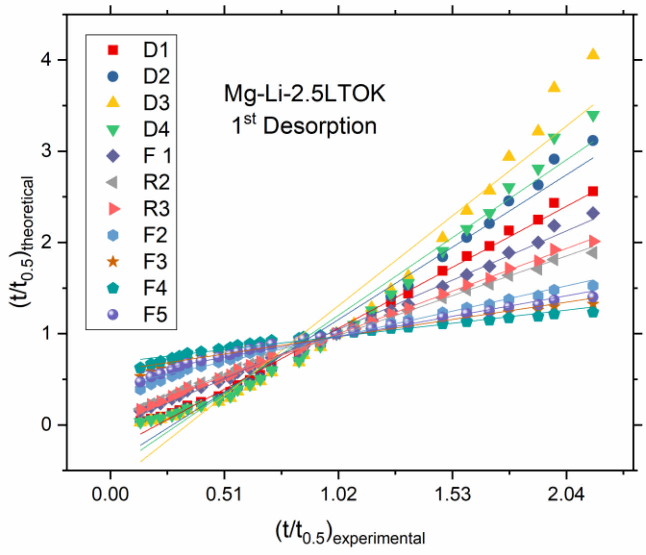

Figure S16 – $(t/t_{0.5})_{experimental}$ vs. $(t/t_{0.5})_{theoretical}$ plot for sample Mg-Li-2.5LTOK at 1st desorption

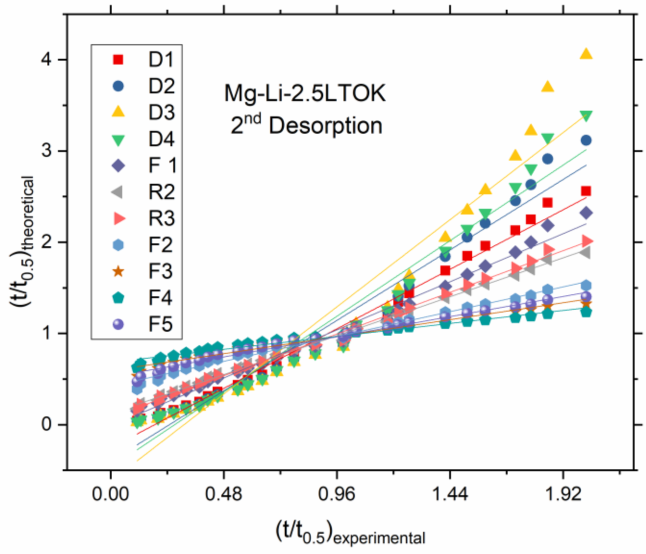

Figure S17 – $(t/t_{0.5})_{experimental}$ vs. $(t/t_{0.5})_{theoretical}$ plot for sample Mg-Li-2.5LTOK at 2nd desorption

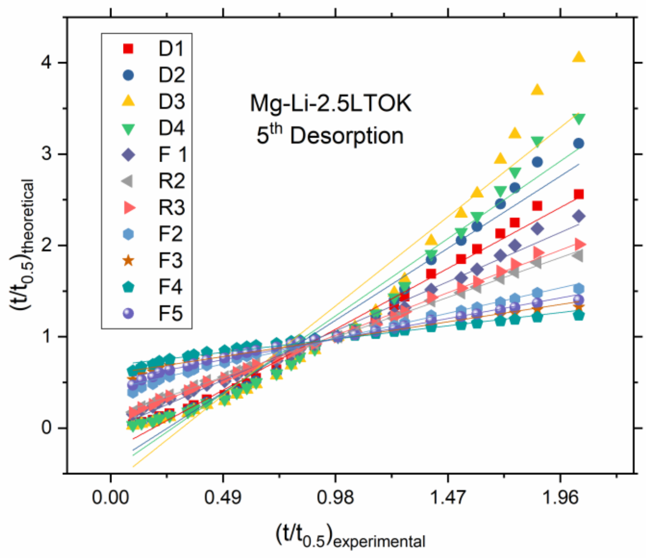

Figure S18 − $(t/t_{0.5})_{experimental}$ vs. $(t/t_{0.5})_{theoretical}$ plot for sample Mg-Li-2.5LTOK at 5th desorption

Table S5 Fitting results for the desorption curves of Mg-Li-2.5LTOK material.

**$Mg(NH_2)_2$+2LiH+0.025LTOK (Mg-Li-2.5LTOK) 1st Des.** Fraction (0.10-0.80)	Intercept Value	Intercept Error	Slope Value	Slope Error	Statistics Adr. R-Square
D1 one-dimensional diffusion	-0.2396	0.02113	1.3283	0.01954	0.99419
D2 two-dimensional diffusion	-0.38732	0.04927	1.5757	0.04557	0.9779
D3 Jander eq. for three dimensional diffusion	-0.60701	0.09841	1.95455	0.09103	0.94456
D4 Ginstling-Braunshtein eq. for three dimensional diffusion	-0.45535	0.06386	1.69223	0.05907	0.96812
F1 JMA - n = 1	-0.00231	0.01712	1.07232	0.01583	0.99415
R2 two - dimensional phase	0.13424	0.0087	0.86564	0.00805	0.99767
R3 three dimensional phase boundary	0.09245	0.00648	0.92764	0.00599	0.99887
F2 JMA - n = 1/2	0.43163	0.01336	0.55057	0.01236	0.98657
F3 JMA - n = 1/3	0.59839	0.01383	0.37646	0.01279	0.96972
F4 JMA - n = 1/4	0.68899	0.01245	0.287	0.01152	0.95826
F5 JMA - n = 2/5	0.52949	0.01412	0.44657	0.01306	0.97742
**$Mg(NH_2)_2$+2LiH+0.025LTOK (Mg-Li-2.5LTOK) 2nd Des.** Fraction (0.10-0.80)	Intercept Value	Intercept Error	Slope Value	Slope Error	Statistics Adr. R-Square
D1 one-dimensional diffusion	-0.25464	0.03255	1.3592	0.03057	0.98652
D2 two-dimensional diffusion	-0.40179	0.06211	1.60861	0.05834	0.96566
D3 Jander eq. for three dimensional diffusion	-0.62039	0.1136	1.9903	0.1067	0.9278
D4 Ginstling-Braunshtein eq. for three dimensional diffusion	-0.4695	0.0774	1.72603	0.07269	0.95422
F1 JMA - n = 1	-0.01499	0.02521	1.09786	0.02368	0.98759
R2 two - dimensional phase	0.12155	0.00924	0.88899	0.00868	0.99743
R3 three dimensional phase boundary	0.07973	0.01155	0.95167	0.01085	0.9965
F2 JMA - n = 1/2	0.42254	0.01139	0.56655	0.01069	0.99047
F3 JMA - n = 1/3	0.59153	0.01212	0.3881	0.01138	0.97728
F4 JMA - n = 1/4	0.68351	0.01109	0.29616	0.01042	0.96763
F5 JMA - n = 2/5	0.52166	0.0122	0.46004	0.01146	0.98352
**$Mg(NH_2)_2$+2LiH+0.025LTOK (Mg-Li-2.5LTOK) 5th Des.** Fraction (0.10-0.80)	Intercept Value	Intercept Error	Slope Value	Slope Error	Statistics Adr. R-Square
D1 one-dimensional diffusion	-0.25222	0.03054	1.36081	0.02875	0.98808
D2 two-dimensional diffusion	-0.39979	0.05947	1.61148	0.05598	0.9684
D3 Jander eq. for three dimensional diffusion	-0.6193	0.11009	1.99538	0.10364	0.93193
D4 Ginstling-Braunshtein eq. for three dimensional diffusion	-0.46776	0.07447	1.72955	0.07011	0.95745
F1 JMA - n = 1	-0.0134	0.02268	1.09956	0.02135	0.98992
R2 two - dimensional phase	0.12336	0.0072	0.88979	0.00678	0.99843
R3 three dimensional phase boundary	0.08149	0.00888	0.95273	0.00836	0.99793
F2 JMA - n = 1/2	0.42369	0.01076	0.56706	0.01013	0.99145
F3 JMA - n = 1/3	0.59237	0.01191	0.38839	0.01122	0.97796
F4 JMA - n = 1/4	0.68417	0.01097	0.29636	0.01033	0.96819
F5 JMA - n = 2/5	0.52263	0.01188	0.46041	0.01118	0.98431

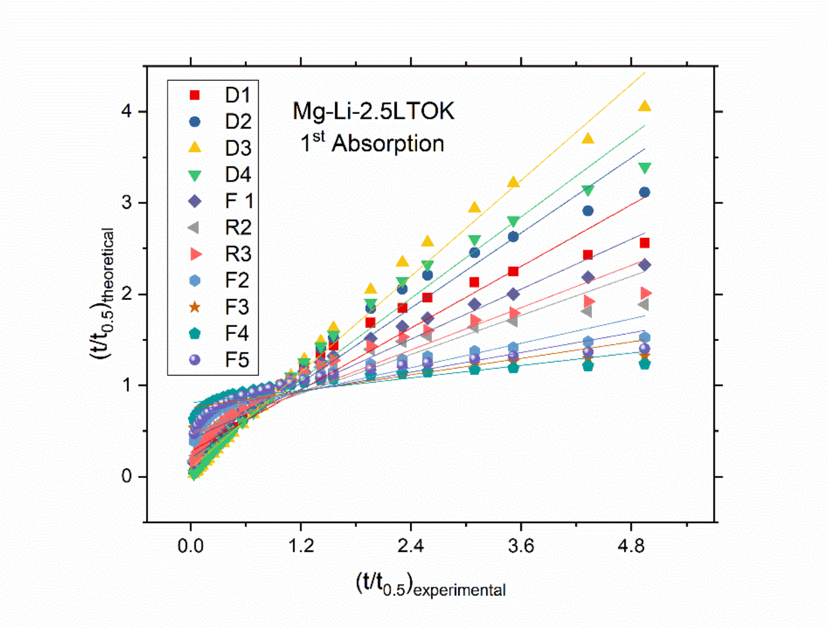

Figure S19 − $(t/t_{0.5})_{experimental}$ vs. $(t/t_{0.5})_{theoretical}$ plot for sample Mg-Li-2.5LTOK at 1st absorption

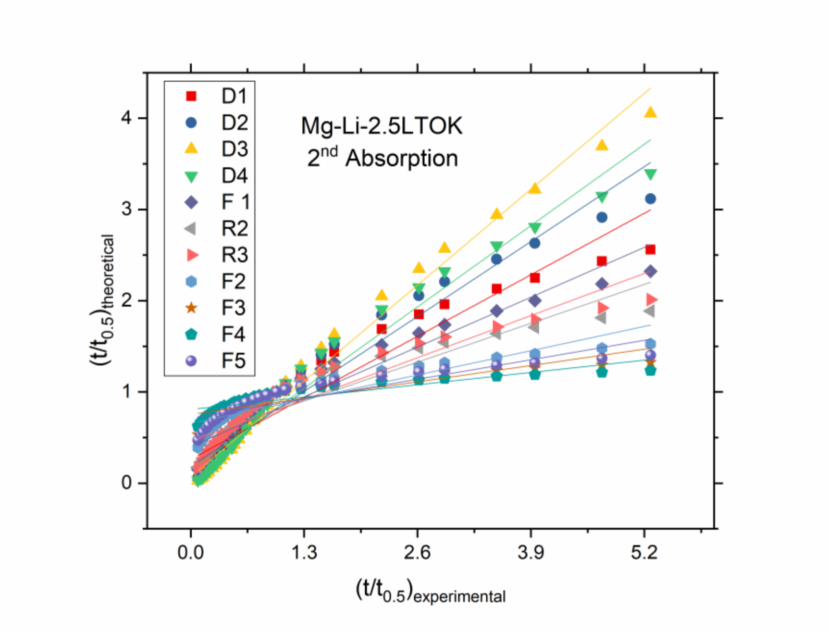

Figure S20 − $(t/t_{0.5})_{experimental}$ vs. $(t/t_{0.5})_{theoretical}$ plot for sample Mg-Li-2.5LTOK at 1st absorption

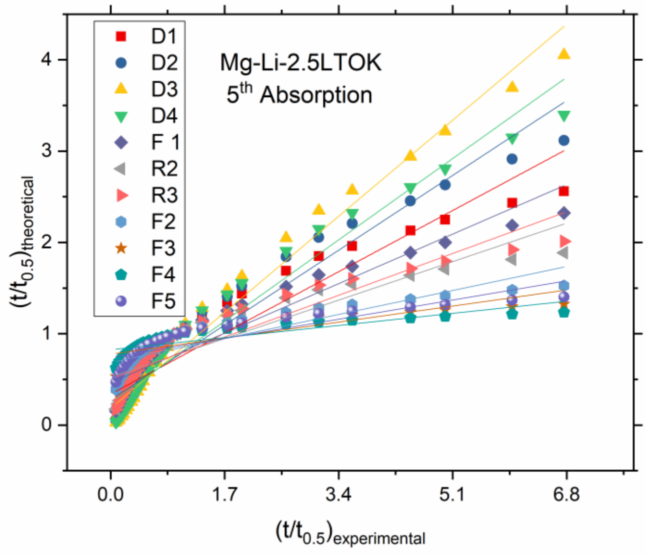

Figure S21 − $(t/t_{0.5})_{experimental}$ vs. $(t/t_{0.5})_{theoretical}$ plot for sample Mg-Li-2.5LTOK at 1st absorption

Table S6 Fitting results for the absorption curves of Mg-Li-2.5LTOK material.

**$Mg(NH_2)_2$+2LiH+0.025LTOK (Mg-Li-2.5LTOK) 1st Abs.** Fraction (0.10-0.80)	Intercept Value	Intercept Error	Slope Value	Slope Error	Statistics Adr. R-Square
D1 one-dimensional diffusion	0.27581	0.05505	0.56387	0.03039	0.92707
D2 two-dimensional diffusion	0.20333	0.04848	0.68579	0.02676	0.96044
D3 Jander eq. for three dimensional diffusion	0.09406	0.03508	0.8764	0.01936	0.98698
D4 Ginstling-Braunshtein eq. for three dimensional diffusion	0.16967	0.04457	0.74409	0.0246	0.9713
F1 JMA - n = 1	0.41169	0.04221	0.4569	0.0233	0.93422
R2 two - dimensional phase	0.4829	0.04665	0.35708	0.02575	0.87629
R3 three dimensional phase boundary	0.46104	0.04556	0.38676	0.02515	0.89711
F2 JMA - n = 1/2	0.65776	0.0341	0.22355	0.01882	0.83836
F3 JMA - n = 1/3	0.75619	0.02648	0.15027	0.01462	0.79498
F4 JMA - n = 1/4	0.81051	0.02144	0.11357	0.01184	0.77131
F5 JMA - n = 2/5	0.71516	0.02988	0.17949	0.0165	0.81299
**$Mg(NH_2)_2$+2LiH+0.025LTOK (Mg-Li-2.5LTOK) 2nd Abs.** Fraction (0.10-0.80)	Intercept Value	Intercept Error	Slope Value	Slope Error	Statistics Adr. R-Square
D1 one-dimensional diffusion	0.26146	0.0579	0.51869	0.02917	0.92108
D2 two-dimensional diffusion	0.18461	0.051	0.63176	0.0257	0.95717
D3 Jander eq. for three dimensional diffusion	0.0687	0.03641	0.8084	0.01834	0.98628
D4 Ginstling-Braunshtein eq. for three dimensional diffusion	0.1489	0.04684	0.6858	0.0236	0.96898
F1 JMA - n = 1	0.40034	0.04488	0.42008	0.02261	0.92726
R2 two - dimensional phase	0.47488	0.04904	0.32767	0.02471	0.86626
R3 three dimensional phase boundary	0.45203	0.04804	0.35515	0.02421	0.88809
F2 JMA - n = 1/2	0.65309	0.03571	0.20489	0.01799	0.82661
F3 JMA - n = 1/3	0.75326	0.02761	0.13756	0.01391	0.78189
F4 JMA - n = 1/4	0.80839	0.02232	0.1039	0.01124	0.75764
F5 JMA - n = 2/5	0.71157	0.03121	0.16439	0.01573	0.8004
**$Mg(NH_2)_2$+2LiH+0.025LTOK (Mg-Li-2.5LTOK) 5th Abs.** Fraction (0.10-0.80)	Intercept Value	Intercept Error	Slope Value	Slope Error	Statistics Adr. R-Square
D1 one-dimensional diffusion	0.3387	0.06242	0.3943	0.02533	0.89936
D2 two-dimensional diffusion	0.27618	0.05714	0.48183	0.02319	0.94101
D3 Jander eq. for three dimensional diffusion	0.18242	0.04526	0.61871	0.01837	0.97674
D4 Ginstling-Braunshtein eq. for three dimensional diffusion	0.24727	0.0538	0.5237	0.02184	0.95509
F1 JMA - n = 1	0.46309	0.04904	0.31922	0.0199	0.90469
R2 two - dimensional phase	0.52565	0.05167	0.24786	0.02097	0.83706
R3 three dimensional phase boundary	0.50638	0.05112	0.26907	0.02074	0.86099
F2 JMA - n = 1/2	0.68555	0.03717	0.15453	0.01508	0.79381
F3 JMA - n = 1/3	0.77554	0.02843	0.10345	0.01154	0.74622
F4 JMA - n = 1/4	0.8254	0.02287	0.07802	0.00928	0.72071
F5 JMA - n = 2/5	0.73795	0.03227	0.12377	0.0131	0.76583
